# Recent Progress in Discovering the Role of Carotenoids and Their Metabolites in Prostatic Physiology and Pathology with a Focus on Prostate Cancer—A Review—Part I: Molecular Mechanisms of Carotenoid Action

**DOI:** 10.3390/antiox10040585

**Published:** 2021-04-10

**Authors:** Joanna Dulińska-Litewka, Yoav Sharoni, Przemysław Hałubiec, Agnieszka Łazarczyk, Oskar Szafrański, James A. McCubrey, Bartosz Gąsiorkiewicz, Piotr Laidler, Torsten Bohn

**Affiliations:** 1 Medical Biochemistry Medical College, Jagiellonian University, 31-034 Cracow, Poland; przemyslawhalubiec@gmail.com (P.H.); agnieszka.lazarczyk@student.uj.edu.pl (A.Ł.); osk.sza2@gmail.com (O.S.); b.gasiorkiewicz@student.uj.edu.pl (B.G.); piotr.laidler@uj.edu.pl (P.L.); 2 Department of Clinical Biochemistry, Faculty of Health Sciences, Ben-Gurion University of the Negev, P.O. Box 653 Beer Sheva, Israel; yoav@bgu.ac.il; 3 Department of Microbiology and Immunology, Brody Medical Sciences Building, East Carolina University, Greenville, NC 27834, USA; mccubreyj@ecu.edu; 4 Nutrition and Health Research Group, Department of Population Health, Luxembourg Institute of Health, 1 A-B, rue Thomas Edison, L-23 1445 Strassen, Luxembourg; torsten.bohn@gmx.ch

**Keywords:** carotenoids, xanthophylls, retinoids, transcription factors, metabolism, nuclear receptors, prostate cancer

## Abstract

Among the vast variety of plant-derived phytochemicals, the group of carotenoids has continuously been investigated in order to optimize their potential application in the area of dietary intervention and medicine. One organ which has been especially targeted in many of these studies and clinical trials is the human prostate. Without doubt, carotenoids (and their endogenous derivatives—retinoids and other apo-carotenoids) are involved in intra- and intercellular signaling, cell growth and differentiation of prostate tissue. Due to the accumulation of new data on the role of different carotenoids such as lycopene (LC) and β-carotene (BC) in prostatic physiology and pathology, the present review aims to cover the past ten years of research in this area. Data from experimental studies are presented in the first part of the review, while epidemiological studies are disclosed and discussed in the second part. The objective of this compilation is to emphasize the present state of knowledge regarding the most potent molecular targets of carotenoids and their main metabolites, as well as to propose promising carotenoid agents for the prevention and treatment of prostatic diseases.

## 1. Introduction

Our knowledge of the role of carotenoids in prostate biology and health has been exponentially growing during the last decades since the first investigations on BC about forty years ago [1]. Despite the increasing amount of data, we still lack not only recommendations for intake of these plant bioactives, but also thorough insight regarding the pathways that are most implicated in the proposed health benefits of carotenoids. This is true particularly for the development of prostate cancer (PC), the most concerning the disease of the prostate in contemporary medicine, although carotenoids have been implicated in other types of cancer such as of the lung [2] and several cardiometabolic diseases [3].

Carotenoids are a fairly diverse group of molecules, derived from many different plant-based food items (tomatoes, carrots, papayas, guavas, watermelons, grapes [4]), as well as some types of fungi and bacteria. Structural and functional similarity exists to retinoids and apo-carotenoids, which are often included in the classification of carotenoids. However, each of these carotenoids presents its own chemical and biological properties, which indicates the need for a separative discussion.

The last decade of studies has shown that our previous view on carotenoids did not entail all the significant aspects. Factors that were not assessed in previous trials, such as the variability of their serum levels (depending on the season), turned out to have strong effects on the outcome. Thus, the latest trials present a paradigm shift in the methodology of the evaluation and standardization of carotenoid-associated health results. A similar revolution has occurred in experimental sciences, which have started to include more sophisticated biological investigations, including microarray analysis, to precisely identify the most potent effectors of carotenoid activity at the molecular level.

These aforementioned issues have inspired us to gather data from the studies investigating the relation between carotenoids and prostate health and to present a comprehensive analysis of their biological activity in this respect. In total, 126 articles have been reviewed—including experimental and epidemiological research—to find answers to the prominent key questions: How do carotenoids modify prostate cell biology? What are the most important biological factors that contribute to the observed in vivo effects of different carotenoids? Which source of carotenoids might be the most promising for the potential treatment of PC?

## 2. Materials and Methods

### 2.1. Search Strategy and Study Selection

We have investigated electronic databases (PubMed, Cochrane, Ovid, National Institute for Health and Clinical Excellence (NICE)).

We decided to extract data between the 1st of January 2009 and 15 November 2020. The following keywords were used for the search: (carotenoids OR lycopene OR carotene OR retinoids OR retinol OR “retinoic acid” OR cryptoxanthin OR astaxanthin OR zeaxanthin OR lutein OR ionone) AND (prostate OR “prostate cancer” OR “prostate carcinoma” OR “prostate physiology” OR “prostate pathology”). The main eligibility criteria were: (a) study investigating the impact of any carotenoid or their metabolites on aspects of prostate physiology and/or pathology; (b) work not being a meta-analysis, review, editorial, comment or duplicate; (c) work published in English.

### 2.2. Data Extraction

The articles were investigated in detail to extract the following data: author, year, evaluated compounds and their concentrations/doses, using cell lines or animal model, quantitative or qualitative results; only results based on carotenoid concentrations (and their metabolites) ≤50 µM were considered, as higher concentrations are clearly never achievable in vivo, even when using pharmacological doses.

The flow chart summarizing the process of data extraction is presented as Figure S1 in Online Supplementary Material.

## 3. Carotenoids—Basic Information

Carotenoids are a group of >1100 pigments synthesized by plants, algae, some types of fungi, and photosynthetic bacteria (Table 1) [5]. Widely distributed in nature, they are responsible for the orange-red color of fruits and vegetables such as tomatoes, oranges and carrots, and the yellow color of various flowers. Carotenoids are present in photosynthetic organelles of all higher plants, mosses, ferns, and algae—they absorb light energy for their use in photosynthesis, and they protect chlorophyll from photodamage [6].

Most carotenoids are 40-carbon terpenoids, with isoprene being their basic structural unit. They can be divided into two main classes: carotenes and xanthophylls. Carotenes contain no oxygen and are unsaturated hydrocarbons. Xanthophylls are yellow pigments with oxygen atoms present in their molecules, e.g., in form of hydroxyl groups [7]. About 50 carotenoids are present in the human diet, while only about 20 can be traced in human blood and tissues [8]. Carotenoids can also be divided according to their provitamin A activity: only those containing a β-ionone moiety can be converted to retinol [9]. The most abundant provitamin A carotenoids are BC, α-carotene and β-cryptoxanthin [10]. The efficiency of their cleavage into vitamin A (VA) is expressed by retinol activity equivalent (RAE) ratios. For example, as 12 µg of BC can be converted into 1 µg of retinol, the RAE ratio for BC equals 12:1.

**Figure 1:**
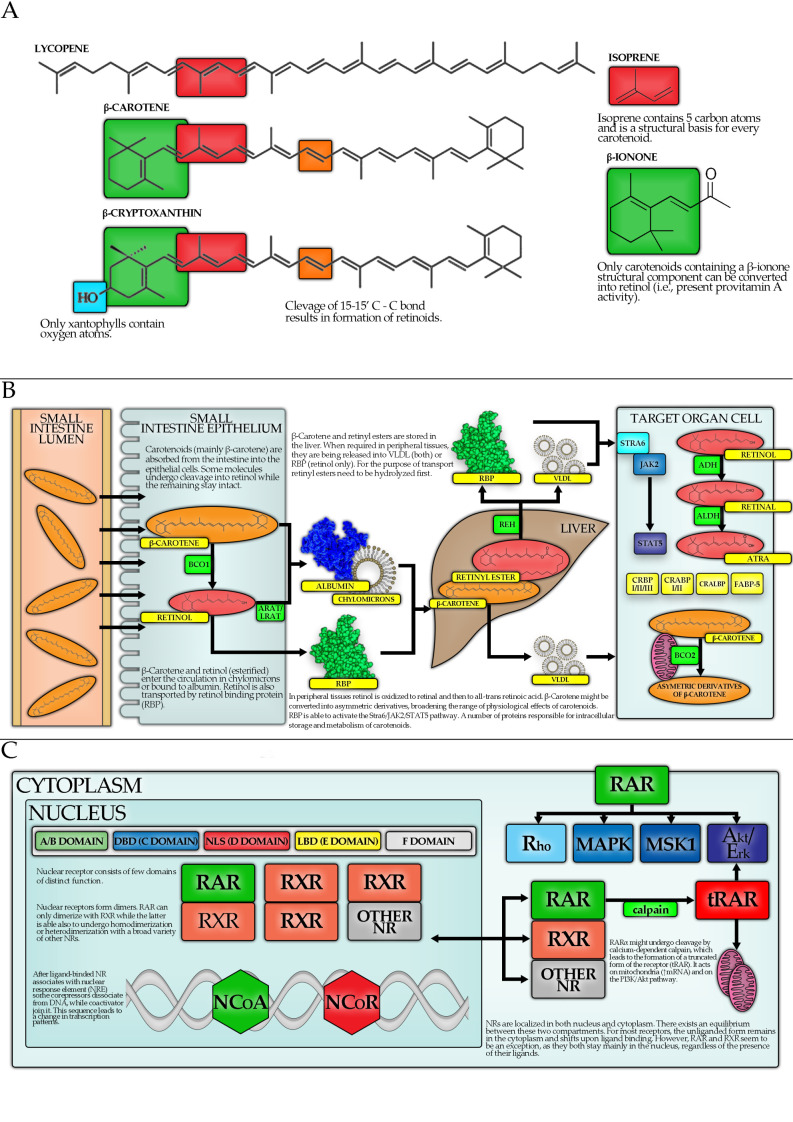
Main metabolic pathway of carotenoids. (**A**) The structure of carotenoids. (**B**) The process of absorption and metabolism of carotenoids. (**C**) The main intracellular targets of carotenoids.

There are two main enzymes engaged in the metabolism of carotenoids within the enterocytes (Figure 1): β-carotene 15, 15′-oxygenase 1 (BCO1) and BC 9′, 10′-oxygenase 2 (BCO2). BCO1 catalyzes the cleavage of provitamin A carotenoids into the retinal. Retinal is then reduced to retinol (VA) or oxidized to all-trans-retinoic acid (ATRA). BCO2 can convert provitamin A carotenoids to apo-carotenoids, however, it has a higher affinity towards non-provitamin A carotenoids. For example, BCO2 converts LC to apo-lycopenal [5]. Major proposed physiological functions of carotenoids in humans include:

1. antioxidant function, e.g., quenching (deactivating) singlet oxygen [11];
2. activation of the nuclear factor E2-related factor 2 (Nrf2)-dependent pathway and thus upregulation of the expression of antioxidant and detoxifying enzymes [12];
3. inhibition of nuclear factor kappa-light-chain-enhancer of activated B cells (NF-κB), in order to prevent its migration into the nucleus, causing a decrease in the production of inflammatory cytokines [12];
4. absorption of blue light by lutein, zeaxanthin, and meso-zeaxanthin in the retina of the eye.

## 4. Carotenoids and Hormones

### 4.1. Introduction

To this date, carotenoids and their derivatives were found to modulate many different endocrine axes, including influences on thyroid hormones, insulin, glucocorticoids, progesterone, estrogens as well as androgens, which is most interesting regarding PC. However, present studies offer inconsistent data on the nature of this interaction and thus it is often difficult to infer a definitive effect of the influence of carotenoids on distinct endocrine regulatory elements.

As mentioned, the crosstalk between carotenoids and androgens was examined. This interaction was possibly conserved during evolution, as in male birds, the carotenoid–testosterone interplay is pivotal for proper integumental coloration development [13]. However, experimental data are highly inconsistent, as carotenoids and their derivatives were shown to either increase [14,15], decrease [16,17,18], or have no effect on serum testosterone [19,20]. A possible explanation for this inconsistency is offered by other studies, showing that the genetic status of BCO1—the enzyme implicated in carotenoid central cleavage—may affect their interaction with testosterone levels in mice [21,22].

Additionally, LC is present at nanomolar concentrations in human semen, often bound to prostasomes. Prostasomes are multilayered vesicles secreted by acinar cells, composed mainly of fatty acids, cholesterol, and sphingomyelin. There is a constant exchange of substances between them and the sperm, which makes prostasomes important in the regulation of the sperm environment [23]. Possibly, LC in prostasomes acts as a free radical scavenger. However, more recent studies which would cover this subject are lacking.

Some carotenoids were also linked to the improvement of insulin-resistance and low-density lipoprotein (LDL) decrease [24,25,26]. Of note, high-density lipoproteins (HDLs) and LDLs are implicated in carotenoid transport in serum and cellular uptake and their relative abundance may affect the biological action of those compounds [27,28].

### 4.2. Carotenoid Metabolism

A recognized classical mechanism of the biological activity of carotenoids involves nuclear receptor (NR) signaling. However, to act as agonists of retinoid X receptors (RXRs) or retinoic acid receptors (RARs), carotenoids must undergo a series of reactions, catalyzed by different enzymes, to be converted into high-affinity ligands, in this case mostly into ATRA. Other metabolites, such as ω3-polyunsaturated fatty acids (ω3-PUFAs) are also potent for receptor binding, although with a lower affinity, whereas some do not necessarily induce its activation upon binding. For example retinal at high concentrations and asymmetric BC cleavage products, which may in fact inhibit NR signaling [29].

Following cellular uptake, retinol is converted into retinal by alcohol dehydrogenase (ADH) and short-chain dehydrogenase (SDR), and then into active ATRA by aldehyde dehydrogenase (ALDH). Apart from that, cytochrome B1 (CYPB1) is capable of converting retinol into retinal or directly into ATRA [29]. BC may enter this pathway after undergoing central oxidative cleavage by cytosolic BCO1 to form the retinal. Another enzyme, BCO2, residing within the mitochondria, is implicated in oxidative but eccentric cleavage of BC, generating other biologically active compounds [29]. Importantly, these products were shown to inhibit RXRα, RARs, peroxisome proliferator-activated receptor α (PPARα), PPARγ and PPARδ activation, as well as inducing growth inhibition in MCF-7 and Hs578T breast cancer cell lines [30,31,32,33,34].

BCO2 is suggested to play a physiological role in the degradation of excess carotenoids to prevent oxidative stress [30]. BCO1 differs in carotenoid affinity, thus partly explaining their different biological activity [33]. Moreover, in humans, BCO1 polymorphism was suggested to affect the biological effects of carotenoids [35]. Furthermore, in BCO1-knock-out mice, a compensatory upregulation of BCO2 was noticed, which was shown to affect LC treatment, as LC caused a significant serum and testicular testosterone level decrease [21]. Apart from that, ALDH distribution was also linked to the regulation of retinoid signaling in embryonic development, as a complex pattern of different ALDH form expression is found in embryos and the perturbation in this system may be lethal [29].

Carotenoids may also impact cell biology directly without being metabolized. These effects include gap junction regulation [36] and oxidative/antioxidant balance influence. In the case of the latter, carotenoids were shown to possess both antioxidant and pro-oxidant properties. The balance between these two actions is affected by carotenoid concentration, where treatment with high doses of carotenoids induced prooxidant effects as opposed to antioxidant properties of low dosage in vitro, as demonstrated for BC and LC in cultures of HT29 colon cancer and murine macrophage-like cell lines [36]. In addition, direct oxidative degradation of BC in the course of antioxidant action may give rise to carotenoid cleavage products (CCPs) that in turn increase oxidative stress by impairing mitochondrial function [30,37,38,39]. Another study provided evidence that oxygen availability may affect carotenoid action, as in cell-based systems, LC resulted in total protection from exposure to high energy radiation at 0% compared to no such effect with a 100% oxygen atmosphere [40]. Interestingly, manganese superoxide dismutase (Mn-SOD) polymorphism was linked to differences in cancer risk reduction due to higher serum carotenoid concentration, as observed in a human observational study [41], also pointing out to differential effects of carotenoids being related to the presence of reactive oxygen species. Distinct carotenoids may also differ in antioxidant properties due to varying relative lipid/water partition coefficients and, therefore, differences in intracellular distribution [36]. BC, residing in lipid membranes, was suggested to influence their properties, which may also be important for cellular fate [39].

### 4.3. Carotenoids, Nuclear Receptors and Transcription Factors

NRs are thought to be the largest group of transcription factors (TFs), capable of changing the expression of multiple target genes, thus playing a vital part in cellular homeostasis regulation. Although most NRs are ligand-activated, in some cases for the so-called “nuclear orphan receptors” (NORs) the ligand is yet to be identified or the receptor activity is regulated at a different level, for instance via post-translational modifications (PTMs) [42,43]. All of the below discussed NRs, except for retinoid orphan receptor α (RORα), β and γ (referred to also as NR1F1, NR1F2, NR1F3) undergo homo- or hetero-dimerization to facilitate their action as TFs [42]. From the point of view of carotenoid-mediated signaling, the most important NRs are RXRs and RARs, with the ligand being VA and its derivatives. In addition, one of the NORs—RORβ (NR1F2), sharing a similarity with RARs—was demonstrated to present a strong affinity for ATRA. At last, PPARδ, an NR implicated in the regulation of fatty acid oxidation and adipocyte differentiation, was also shown to be potent for retinoid-binding [42].

RXRs and RARs are two families of NRs, each consisting of three proteins encoded by different genes: RARα, β, γ (NR1B1, NR1B2, NR1B3) and RXRα, β, γ (NR2B1, NR2B2, NR2B3), respectively. Unlike some steroid receptors, both RXRs and RARs are believed to be localized mainly in the nucleus, independently of ligand presence—although they undergo constant nucleo-cytoplasmic shuttling, reaching a balance established by the availability of both ligand and dimerizing partners [42]. To actively regulate transcription of genes, both RXRs and RARs need to undergo dimerization with other NR, enabling them to bind specified regions in the promoter or enhancer regions of target genes, termed Retinoid X Receptor Responsive Elements (RXREs) and Retinoic Acid Receptor Responsive Elements (RAREs), respectively. Whereas RARs may act only as heterodimers with RXRs, the second may undergo homodimerization or heterodimerize with a member of many other NR families, such as RAR, liver X receptor (LXR), constitutive androgen receptor (CAR), farnesoid X receptor (FXR), PPAR, hepatocyte nuclear factor 4 (HNF4), Nr2f, vitamin D receptor (VDR), nuclear receptor-related 1 protein (Nurr1), pregnane X receptor (PXR) and triiodothyronine receptor (TR3) [42,44].

The majority of RAREs do not seem to be directly involved in gene expression regulation via the classical mechanism, suggesting other possible roles for RXRs and RARs in gene expression regulation, for instance by affecting deoxyribonucleic acid (DNA) structural changes (loop forming etc.) or contribution to the formation of other protein complexes [42]. Furthermore, complexity is reached as different heterodimers of RXR are thought to be differently dependent on ligand binding. This led to the classification of heterodimers into three classes: nonpermissive, permissive, and conditionally permissive (Table 2). There is increasing evidence that the type of reaction may also depend on the cell type and availability of cofactors [42,44]. Signaling termination may be mediated by ligand-bound receptor phosphorylation and subsequent ubiquitination, followed by proteasomal degradation.

Given the role of androgen signaling in PC, it is important to understand its complex crosstalk with retinoid receptors. RXRα physically interacts with unliganded androgen receptor (AR) to act as a weak co-activator. However, RXRα diminishes dihydrotestosterone -mediated gene expression. On the other hand, independently of androgen presence, AR is thought to repress RXR transcriptional activity [45]. Moreover, RXR interaction with orphan NR CAR was also described to diminish the activity of the latter [44]. Furthermore, putative androgen-responsive element (ARE) was found in the RARα gene promoter, suggesting androgens may directly regulate its expression [46]. Of note, one study found that, upon prostate tumorigenesis, upregulation of RAR with subsequent downregulation of AR took place. This perturbation of a balance between AR and RAR coexisted with the inability of ATRA to induce cell proliferation in cancer cells, as it did in normal ones [47]. Conversely, stable expression of full-length AR in an AR-null PC-3 cell line was even shown to sensitize cells for retinoid inhibitory action [48].

However, it is also important to emphasize that many biological effects of carotenoids are thought to be independent of NR activation [42]. Research has highlighted the role of another TF, although not belonging to the NR superfamily, in mediating the biological action of carotenoids. The nuclear factor erythroid 2-related factor 2 encoded by Nuclear Factor, Erythroid 2 Like 2 (NFE2L2) gene is a basic leucine zipper (bZIP) protein, regarded as a master regulator of cellular antioxidative response [49]. Upon nuclear translocation, it binds to the antioxidant responsive element (AnRE) or electrophile-response element (EpRE) in the DNA to regulate the transcription of multiple target genes, such as NAD(P)H quinone oxidoreductase, glutamate-cysteine ligase, thioredoxin reductase 1, heme oxygenase-1 (HMOX-1), glutathione S-transferase, UDP-glucuronosyltransferase and multidrug resistance-associated proteins implicated predominantly in antioxidative response and xenobiotic metabolism [49]. Its physiological role, however, encompasses actions far beyond reducing oxidative and xenobiotic stress, including reducing inflammatory response, regulating autophagy, mitochondrial function, and cellular metabolism [49]. Mechanistically, for transcriptional activity, Nrf2 needs to dimerize with one of the small musculoaponeurotic fibrosarcoma (sMaf) proteins, bind to AnRE and recruit co-activators and nucleosome-remodeling complexes to facilitate RNA polymerase II-dependent transcription [49]. As Nrf2 messenger ribonucleic acid (mRNA) is constitutively expressed, most of its regulation occurs at the protein level. When synthetized in the cytosol, Nrf2 is abruptly sequestered by the kelch-like ECH-associated protein 1 (Keap1) homodimer, ultimately facilitating proteasomal degradation of Nrf2. Electrophilic or oxidative stress causes covalent modification of cysteine residues in Keap1, abrogating Keap1-Cul3-Nrf2 interaction, thus stabilizing the latter, facilitating its accumulation and nuclear translocation [49]. Interestingly, the results of in vitro studies suggest that Nrf2 regulation may also occur at the epigenetic level, via close regulation by micro RNAs (miRNAs) or DNA methylation [50]. Nrf2 was also shown to interact with the ATRA-RARα complex, which results in comprised AnRE binding and transcriptional activity of the first [50]. Unliganded RARα was also shown to bind Nrf2 at a different site, resulting in Nrf2 inhibition [50].

Therefore, Nrf2 in cancer biology may act as a tumor suppressor during initiation and promotion of carcinogenesis and conversely as an oncogene at late stages. Consistently, this ambiguity is reflected in PC biology. Enhanced Nrf2 signaling due to hypermethylation of Keap1 promoter or mutation of Keap1 or Nrf2 gene were reported in PC [50]. Conversely, in Transgenic Adenocarcinoma Mouse Prostate (TRAMP) mice, PC cells were characterized by hypermethylation of the Nrf2 promoter, resulting in a decrease in its activity [50]. Interestingly, a recent paper reported reactive oxygen species (ROS)-independent Nrf2 activation as a result of PC, which depended on endoplasmic reticulum-stress mediated GRP78/BiP translocation to the cell surface [51]. Importantly, Nrf2 was shown to be responsive to carotenoid regulation. LC, BC, phytoene as well as astaxanthin (AST) mediated Nrf2 nuclear translocation and enhanced Nrf2 target gene transcription [52]. However, carotenoids are hydrophobic, raising the question of whether Nrf2 is rather activated by their derivatives. Indeed, it is suggested that an α,β-unsaturated carbonyl group is required for the reaction with Keap1 and subsequent Nrf2 release and activation [53]. This property is characteristic only for xanthophylls such as AST, whereas other carotenoids are incapable of Nrf2 induction [53]. Furthermore, oxidation products of BCO1- and BCO2-mediated carotenoid metabolism such as apocarotenals or diapocarotenedials, as well as some derivatives of enzymatic cleavage by the 9′, 10′-monooxygenases, are potential candidates for direct activation of Nrf2 [53]. This again highlights the importance of carotenoid metabolism in their final biological action, as discussed in Section 5.

## 5. Molecular Mechanisms of Carotenoids—Action Related to PC

It has to be emphasized that in vitro studies on cell cultures should be interpreted with particular caution. Generally, carotenoids can be easily oxidized and are prone to degradation, induced by heat or light. Oxidation can be initiated by numerous oxidizing agents, including atmospheric oxygen. Oxidized carotenoids may further react with themselves or other chemical compounds within the cell culture to form a plethora of products. High temperature enhances the oxidation rate of BC. Thermal processing is also likely to cause the breakdown of the cellular matrix of the plant material and may also induce trans to cis isomerization of carotenoids, e.g., LC. Finally, light exposure also degrades carotenoids by several proposed mechanisms via photooxidation [54]. Therefore, the interpretation of cell-culture studies may pose a challenge as the observed results can be caused by the oxidized products rather than the parent compounds.

Another important issue to take into account when interpreting cell-culture-based findings is differences between the concentrations of carotenoids present in body tissues and those used in cell culture. According to the literature data, LC levels reach 700 nM in the prostate and approximately 384–740 nM in the serum, mean BC concentration in the prostate tissue reaches 600 nM and in the serum about 360–874 nM [55]. Endogenous ATRA in human plasma, quantified by liquid chromatography-tandem mass spectrometry approach, is even lower, within the range of 1.9 to 9.2 nM [56]. Interestingly, in part II of our review [57], the highest observed concentrations in participants of observational and interventional studies were 1.2 µM for LC and 6.7 µmol/L for BC. In one human study discussed below, the excessive supplementation raised serum levels of LC to 14.5 µM [58], while in the Alpha-Tocopherol, Beta-Carotene Cancer Prevention (ATBC) study, after 3 years of supplementation of 20 mg BC per day, the 80th percentile for BC concentration was 8.4 µM [59]. In cell culture studies, often much higher concentrations of carotenoids have been employed, up to 10 µM or even higher, and thus results should be interpreted with care. On the other hand, exposure times in cell-culture studies are typically short (several hours), and thus some effects may also be underestimated. In the present review, we have not considered concentrations higher than 50 µM. This level was chosen as many studies examine concentrations that include, among lower ones, also concentrations up to 50 µM.

Bearing these general considerations in mind, cell study-based findings should not be discarded as irrelevant, as they still can yield interesting mechanistic findings, and are useful for hypothesis building. The above-mentioned issues are merely inherent limitations of most cell-culture-based experiments.

In addition, there are certain points to consider when interpreting studies using whole food extracts containing carotenoids, as these extracts are likely to contain a number of additional lipophilic compounds such as vitamin E, or carotenoid breakdown products, and are thus far from pure. In addition, the efficiency of carotenoid absorption is affected by several matrix factors (e.g., presence of fiber, minerals, fat, and fat-soluble micronutrients). Carotenoid bioavailability is also restricted or enhanced by host-related factors such as diseases, lifestyle habits, gender, and age, as well as genetic variations including single nucleotide polymorphisms (SNPs) [60]. Moreover, carotenoid absorption depends on, among others, food processing, meal composition, the activity of digestive enzymes and transport efficiency across the enterocyte, as reviewed previously [61].

**Figure 2:**
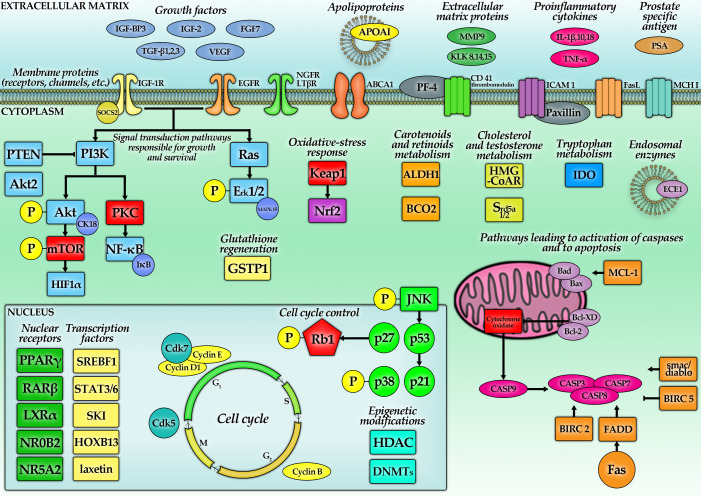
The molecular pathways of carotenoid action in PC (discussed in Section 5), which have been the subject of research in the last decade. They involve the growth factor receptors (i.e., epidermal growth factor receptor (EGFR) and insulin-like growth factor receptor (IGFR)), ABC transporters, molecules of intracellular adhesion (i.e., ICAM-1). Moreover, a variety of proteins, which control the cell cycle and process of apoptosis, change their level under the influence of carotenoids. Significant changes were discovered, as well in the components of extracellular matrix (i.e., matrix metalloproteases (MMPs) and kallikreins (KLKs)). More detailed information of given carotenoids on the particular factors from this figure is shown in Table 4 (the factors marked with red color are not directly reviewed in our work; however they are necessary to understand their relation to well-established functions, e.g., mTOR).

A summary of the findings of this section is presented in Table 3 and Table 4 as well as Figure 2.

**Table 3:** Overview of results of laboratory studies investigating the impact of carotenoids on prostate cancer (PC) (cell lines, PC tissue or mice model).

Carotenoid	Investigated Feature	Concentration Range or Dose Used/Type of Food Extract Used	Investigated Entity	Results	Commentary	Reference
Lycopene	Growth inhibitory effect ^1^	1 µM (1–100 µM) ±1 nM docetaxel (d) or ±10–25 µM temozolomide (t) for 72 h (12–96 h)	LNCaP LAPC-4 DU145 PC-3 22Rv1	5%↓ (+d: 21%↓), IC_50_ = 2 µM 19%↓ (+d: N/C) 24%↓ (+d: 78%↓), IC_50_ = 3 µM N/C (+d: 21%↓, +t: 70%↓) or 25%↑ (48 h), IC_50_ = 4 µM 10%↓ (+d: 20%↓)	For 10 µM, the peak inhibitory effect was shown in LNCaP, Du145 and PC-3. One study found an increase of PC-3 proliferation after 1 µM or 5 µM LC. Algal LC 20–50 µM caused ~50–60%, while tomato LC caused ~40–50% growth reduction in PC-3. LC extracted from natural sourced induced a ~40–50% reduction in PC growth.	[62,63,64,65,66,67,68,69,70,71,72]
	Apoptosis	10 µM for 48–96 h for 96 h	DU145 PC-3 primary PC cells	↑ (5 x, 96 h) ↑ (2.2 x, 48 h) ↑ (1.35 x, 48 h) ↑ (2.25 x, 96 h)	Tomato paste, extract and sauce induced an average 51-fold increase in the apoptotic rate in PC cells, yielding a more pronounced result than addition of pure LC.	[68]
		500–5000 µg/mL in form of tomato paste, extract, etc.	BPH cells	N/C		[70]
		TNF-α + tomato extract 2 g/mL (doses 0.5–15 mg/ml) for 6 h	LNCaP DU145	↑ apoptosis		[73]
		[LC concentration in the extract 0.9–8.6 µM]	PC-3	Fas↑, CASP9↑, HIF1α↑		[74]
	Colony formation	20 µM and 50 µM for 12 h	PC-3 DU145	110 colonies (20 µM), 59 colonies (50 µM) and 180 colonies (untreated) 76 colonies (20 µM), 35 colonies (50 µM) and 115 colonies (untreated)	LC reduced colony formation in PC-3 and DU145.	[69]
	Cellular accumulation	1 μM and 3 μM for 24 h	PrEC LNCaP C4-2 DU145 PC-3	150 and 250 pmol/10^6^ cells 75 and 200 pmol/10^6^ cells 10 and 50 pmol/10^6^ cells 20 and 80 pmol/10^6^ cells 60 and 100 pmol/10^6^ cells	PC cells tended to accumulate less LC than healthy prostatic tissue.	[62]
	Cellular adhesion and metastatic potential	1.15 and 2.3 µM for 24 h 2 g/mL tomato extract (doses 0.5–15 mg/mL) for 6 h	PC-3 DU145 PNT2	↓ adhesion	Inhibition of adhesion was most pronounced for DU145 cells.	[75]
		[LC concentration in the extract 0.9–8.6 µM]	PC-3	ICAM1↓, MMP9↓		[74]
	Cholesterol metabolism pathways	2.5–10 µM for 12–48 h	LNCaP DU145 PC-3	↓cell cholesterol HMG-CoAR↓ ApoAI↑ PPARγ↑, LXRα↑, ABCA1↑	Inhibition of cholesterol synthesis affected small G proteins like Ras, which require farnesylation. Cells with mutated, less stable Ras proteins were 2–3 times more prone to the LC treatment. Addition of siRNA targeted to LXRα decreased the effects of LC (caused an increase in cells’ proliferation). LC augments anti-proliferative effect of PPARγ agonists in PC	[64,65,76,77]
		25 μM + 25 μM ciglitazone for 48 h	PC-3	↓ 82% cell proliferation		[67]
		10 µM for 24 h	PC-3	↓HMG-CoA		[77]
	ROS, NF-κB effectors, Akt	0.5–20 μM for 96 h	LNCaP	↓ROS, NF-κB↓		[68]
		2.5–10 µM for 24 h	LNCaP	↓cyclin D1, ↓Bcl-2, ↓Bcl-XD, ↓p-Akt p53↑, p27↑, p21↑, Bax↑		[77]
		Tomato paste (30.9 mg LC/kg feed) for 10 days	male NMRI nude mice	↓ NF-κB activity STAT3↑, STAT6↑	PC3-κB-luc cells were injected into tomato-fed and control mice; no difference in tumor growth, reduced NF-κB in tomato-fed mice.	[74]
		10, 25 and 50 µM for 48 h	PC-3	↓Akt2 ↑miR-let-7f	miR-let-7f targeted Akt2 mRNA	[78]
	Cell cycle, pro- and antiapoptotic proteins	9 and 18 mg LC/kg of feed for 7 weeks	BALB/c nude mice	↓tumor volume	The tumors in mice were induced by PC-3 cell injection.	[79]
		1 µM, 2 µM and 4 µM for 24–72 h	PC cells from Gleason score 6 tumor	Bcl2↓ Bax↑ IGF-1↑	No change was observed in the group treated with the 1 µM solution.	[80]
		0.5–5 μM for 72 h	PC-3	↓TNF-α		[72]
	Gene methylation, GSTP1, IGF-1	1 μM, 2 μM, and 4 μM for 7 days	LNCaP	N/C GSTP1 N/C GSTP1 promoter methylation	LC did not have an influence on the demethylation of the GSTP1 gene promoter.	[81]
		1 μM and 10 μM for 72 h	LNCaP LNCaP/IGF-IR	IC_50_ for LNCaP—36 µM IC_50_ for LNCaP/IGF-IR—0.08 µM	LNCaP/IGF-IR were 400-fold more susceptible to LC treatment. LC hypothetically interfered with the activation of IGF-IR.	[63]
	BCO1 and BCO2	5-aza-2dC (methyltransferase inhibitor) + (in the next step) 1 μM LC for 24 h	LNCaP DU145	BCO2↑ in LNCaP BCO2 N/C in DU145	BCO2 levels supposedly decreased during the PC progression. Overexpression of BCO2 potentialized the antiproliferative effects of LC.	[62]
	Mice model studies on IGF-I pathway and use of lycopene along with cytotoxic agents	28 mg LC/kg of feed a day in tomato powder (TP) or lycopene beadlets (LB) for 20 weeks	TRAMP mouse model	↓ incidence of PC: 60% (LB) vs. 95% (control), *p* = 0.0197; N/C in IGF-I and IGF-BP3 in all groups	30% of the LB-fed mice developed BPH, unobserved in other groups.	[82]
		placebo beadlets, tomato powder (providing 384 mg LC/kg diet) and LC beadlets (providing 462 mg LC/kg diet)for 4 weeks	TRAMP mouse model	5α-reductase isoforms↓ (Srd5a1, Srd5a2) androgen receptor co-regulators↓ (Pxn and Srebf1) and changes in 30 other genes’ expression	LC interfered directly with androgen signaling.	[18]
		50 mg LC for 42 weeks +800 IU vit. E and 200 μg seleno-DL methionine for 42 weeks [control group with no supplementation]	Lady transgenic mouse (12T-10)	PC development frequency: 90% (control) 15% (suppl.) PF4↑ and CD41↑ (suppl.)	The authors suggested that PF-4 blocked angiogenesis at early stage of tumorigenesis.	[83]
		Several groups: LC (15 mg/kg daily) low dose docetaxel (5 mg/kg per week) ± LC high dose docetaxel (10 mg/kg per week) ± LC for 15–74 days	NCR-nu/nu (nude) mice	↓ tumor growth dynamics ↑38% in docetaxel’s inhibitory effect on tumor growth	DU145 cells were used to induce tumors in mice. The LC supplement in combination with the lower dose of docetaxel had the same efficacy in prolonging the life of mice as the higher dose of docetaxel. The mice were observed until the tumor reached V = 1500 mm^3^ or when a mouse died spontaneously.	[63]
ATRA	Growth inhibitory effect	10–160 nM for 72 h	PC-3 DU145	8–62%↓ 11–65%↓		[84]
		80 nM ± 40 µM zolendronic acid (z) for 72 h 40 nM ± 20 µM zolendronic acid (z) for 72 h	PC-3 DU145	39%↓ (+z: 75%↓) 23%↓ (+z: 60%↓)		
		10 µM ±leucine ± β-alanine ±RARα antagonist (Ro415253) for 48 h	LNCaP	32%↓	Different types of ATRA conjugated with leucine or β-alanine caused similar reduction in cell number, however Ro415253 enhanced effectiveness of conjugates (but not ATRA).	[85]
		10 µM ± spermine (s) conjugated (RASP) for 24 h	LNCaP PC-3	10%↓ (+s: 50%↓) 10%↓ (+s: 70%↓)		[86]
		10 nM for 5 days	PC-3, LNCaP, DU145	the minimal concentration capable of (at any degree) reducing PC cell growth in vitro	ATRA main mechanism of action was apoptosis induction, while RASP caused necrosis.	[87]
	Protein level	80 nM ± 40 µM zolendronic acid (z) for 72 h	PC-3 DU145	CASP3, 7↑, TNRSF↑, BIRC2↓, BIRC5↓, MCL-1↓, LTβR↓, Bad↑, Bax↑, Fas↑, FADD↑, smac/diablo↑, Bcl-2↓, p53↓		[84]
		20–120 µM for 24–72 h	DU145	HOXB13↑ (achieved for 20–50 µM)	Methylases DNMTb3 and EZH2 were downregulated by ATRA, which resulted in activation of HOXB13 promoter.	[88]
		2 µM ± caffeic acid phenethyl ester for 24 h	PC-3, LNCaP, DU145, PrEC	thrombomodulin ↑ (except for DU145)		[89]
		1 µM ± spermine (s) conjugated (RASP) for 24 h	PC-3	RARβ↑	siRNA targeting RARα eliminated RARβ expression in PC-3 cells and impeded ATRA (or RASP) based effects.	[86]
		1 µM for 24 h	TAMs incubated in serum derived from PC-3 culture	IL-1β↓, IL-10↓, IDO↓, VEGF↓, MHC I↑, FasL↑, NF-κB↓	Though ATRA reduced TAMs proliferation, their viability and functioning were not impaired significantly.	[90]
		0.1 µM or 1 µM ± 1 µM rosocovitine (r) for 24 h	DU145	Cdk5↑, p27 N/C (+r: ↑)		[91]
		1 µM for 72 h	LNCaP	Laxetin↑	The results strongly suggested that ATRA induced cell cycle arrest in G1 phase.	[92]
Retinol	Cellular adhesion, metastatic potential	10 µM for 72 h	PC-3	13%↓ adhesion	Retinol possessed stronger anti-adhesive activity and antiproliferative than ATRA, reducing adhesion by 23% and growth by 79% in 10 µM concentration.	[93]
Vitamin A (ATRA + retinene + retinal + retinol)	Growth inhibitory effect Gene expression	5–15 µM for 24–96 h 5–15 µM ± 10 µM vitamin D for 24–72 h	PC-3	5–25%↓ Bax↑, cyclin D1↓	The study found that VA + VD combined reduce mitochondrial transmembrane potential. Synergistic effects were suggested.	[94]
β-Carotene	Growth inhibitory effect	0–6 µM for 72 h	LNCaP, DU145 PC-3	N/C IC_50_ = 13.0 ± 2.6 μM	IC_50_ could not have been estimated. Incubation with 6.5 μM BC decreased the activity of an AR-luciferase construct in LNCaP cells by about 40%, however it did not influence PSA secretion. CI for combination with LC was 0.65.	[71]
		1–5 µM for 12 h 20 µM for 12 h	PC-3	5%↑ 20↓		[95]
	Protein level	1–5 µM for 12 h	PC-3	VEGF↑ (3 ×)	For a range of 5–10 μM, the effect was weaker and regardless of the initial concentration diminished after 6 h.	[95]

^1^ Here, due to the large amount of studies the values are given in X (Y–Z) format while the Results column refers to the X value (as long as it is not otherwise specified), which is concerned to be the most representative, i.e., used most commonly along the studies, while (Y–Z) describes the range of concentrations or doses, or time intervals which were overall used and are discussed in more detail in Commentary column or in the main text. Arrow up (↑) means that given entity (i.e., cell growth, apoptosis, protein concentration, or gene expression) increased (if specified, by N%, or N-times (N x)), while arrow down (↓) refers to its decrease in the analogous way.

### 5.1. Lycopene—Studies with Cellular Models

The PC cell lines most commonly used in cell culture studies are LNCaP, DU145 and PC-3. Molecular characteristics of those cell lines have already been thoroughly analyzed [99]. Generally, the results of studies should be interpreted in the context of the characteristics of the cell lines used, as they represent different stages of carcinoma development. The LNCaP cell line was established from a lymph node metastasis of a human prostate adenocarcinoma [100]. The cells are IFN-resistant and possess high-affinity, specific ARs in the cytosol and nuclear fractions. In vitro, 5α-dihydrotestosterone (DHT) modulates cell growth and stimulates acid phosphatase production. The DU145 cells are derived from a brain metastasis of primary PC [101]. They express ARs, but are hormone-irresponsive (5α-DHT does not modulate their growth) and do not express PSA. Their ability to accumulate LC is smaller than that of both LNCaP and PC-3, which requires consideration. PC-3 cells are derived from bone metastasis of prostate adenocarcinoma [102]. PC-3 cells do not express PSA and their proliferation is insensitive to androgens. It is suggested that they are a model of prostatic small cell neuroendocrine carcinoma (SCNC) [103].

#### 5.1.1. Growth-Inhibitory Effects

Chemotherapeutic drug treatment results in various detrimental side effects and improvements in therapy need to be developed. Docetaxel is one of the most important chemotherapeutics, which is used to treat castration-resistant prostate cancer (CRPC). In one study, 22Rv1, LNCaP, LAPC-4, DU145, and PC-3 cells were treated with 1 nM docetaxel, 1 μM LC and both compounds for 3 days. Growth of 22Rv1, LNCaP, LAPC-4, PC-3, and DU145 were inhibited by 1 nM docetaxel by approximately 54%, 35%, 19%, 27%, and 0%, whereas 1 μM LC decreased their growth by approximately 10%, 5%, 19%, 0% and 24% [63]. These results are partially opposite from another study, which found a reduction only in LNCaP cell number, but no change in the amount of DU145, C4-2 and PC-3 cells for the same LC conditions [62]. Still, synergy was shown, as a combination of docetaxel and LC reduced cell growth by 78% (DU145), 21% (PC-3 and LNCaP), 20% (22Rv1), and 0% (LAPC-4). Thus, the results demonstrated an increase in growth-inhibitory effects of these combinations by approximately 38% when compared to treatment with docetaxel alone [63].

In another study, DU145 cells were treated with LC with concentrations ranging from 2 to 20 µM for 12 h, 24 h and 48 h. Interestingly, LC reduced cellular growth in a dose-dependent manner by up to 10 µM, followed by a decrease of the inhibitory effect at 20 µM. A similar result was obtained for PC-3 and LNCaP cell lines [64,65]. In another study, it was shown that proliferation of PC-3 cells increased after 48 h treatment with 1 µM (23%) and 5 µM (18%) of LC. At higher concentrations (10 µM and 25 µM) though, LC gave comparable results to the control. However, such concentrations are not physiological, apart from the gut. Moreover, without a special emulsifier, such concentrations are technically difficult to achieve [66]. In yet another study, the authors intended to measure the synergy of LC and an alkylating agent—temozolomide. When given alone, it did not have a significant effect on the growth of PC-3, but the combination of 25 µM LC and 10 or 25 µM temozolomide inhibited cell proliferation by 72% and 77%, respectively [67]. Not only in cell lines, but also in primary cells obtained from resected prostate, the effects of LC were investigated. An amount of 1 µM of LC treatment decreased the number of cells, yet the results were not statistically significant. The cells from benign prostatic hyperplasia (BPH) were unaffected by LC at a concentration of 1 µM [68].

It was hypothesized that the efficacy of LC strongly depends on its source of origin, suggesting perhaps additional or synergistic effects, though it is also known that the solubility of LC depends on its source of origin, influencing, e.g., amorphous vs. crystalline state of LC or its bioavailability. PC-3 and DU145 cells were treated with extracts of algal LC (AL—from *Chlorella marina*) and tomato LC (TL). Treatment with 20 and 50 µM AL resulted in 46% and 39% cell viability, respectively, after 24 h incubation. The same concentrations of LC from TL resulted in 64% and 51% PC-3 cell viability. DU145, when treated with the same concentrations of LC from AL, resulted in a greater biological effect than the tomato extract (44% and 32% viability, respectively). TL treatment resulted in 61% (20 µM) and 45% (50 µM) cell viability after 24 h. These results show that the LC extract from algae caused a greater biological effect than the tomato extract [69]. Furthermore, LC extract from algae and tomato had stronger effects on cellular viability than purified LC.

A similar experiment investigated the effects of LC from different sources on PC cells obtained from PC tissue. The extracts included tomato paste (75 μg/g LC), tomato sauce (160 μg/g LC), ketchup (142 μg/g) and tomato extract (81 μg/g). The PCs were incubated with LC extracts, diluted to 5 mg of extract per mL for 96 h. LC from tomato paste caused a 54% reduction in cell growth. LC from tomato extract reduced it by 47%, from tomato sauce by 44%, and from ketchup by 51% [70]. Interestingly, the growth inhibition of LC was stronger than that of phytoene, phytofluene, AST, and BC in the LNCaP cell line. An amount of 0.3 μM of LC did not cause any growth inhibition in LNCaP, while it did at 1 μM. This concentration of LC was found in the blood of people who ate large amounts of tomato products. Studies found also that the half-maximal inhibitory concentration (IC_50_) of LC for LNCaP, DU145 and PC-3 cell lines were 2.0 µM, 3.0 µM, 4.0 µM respectively [71]. Another study showed that the effect of LC on PC-3 cells started at 1.25 µM [72], suggesting that high but still achievable concentrations would be expected to have biological effects. Thus, the inhibitory effect did not change linearly with LC concentrations in the investigated products [70].

#### 5.1.2. Lycopene—Apoptosis, Colony Formation, Cellular Accumulation, Adhesion, and Migration

Induction of apoptosis is an important mechanism of tumor elimination. DU145 and PC-3 cell lines treated with 5 and 10 μM LC for 48 and 96 h presented a significant increase in apoptosis. In DU145, the number of apoptotic cells increased five-folds after 96 h of 10 μM treatment. In PC-3 cells, the highest increase regarding the accumulation of apoptotic cells after 10 μM LC treatment was observed after 48 h of incubation and was equal to 2.2-fold. For primary PC cells, there was a 1.35-fold change after 48 h and a 2.25-fold increase after 96 h. In BPH cells no changes were observed [68]. Even more pronounced were the results from treatments with food-derived tomato extracts, as described above. LC present in processed tomato products is more effective, perhaps as the formation of LC cis-isomers during food processing makes it more bioavailable [73].

Tomato paste, extract and sauce induced on average a 51-fold increase in the apoptotic rate in PC cells. Only for ketchup extract, this response was weaker [70]. Similar effects were obtained in another study, which examined the effects of various tomato-based products on the cell cycle, apoptosis, and proliferation.

LNCaP and DU145 cells were treated with tomato extract, paste, ketchup and sauce at various concentrations (500–5000 µg/mL) for 96 h. All samples showed a decreased survival and increased apoptosis of tumor cells [73]. Not surprisingly, the study that found an increase in cell proliferation after 25 μM of LC treatment, did not observe any impact on apoptosis after its administration. Nevertheless, LC influenced apoptotic cell rate synergistically with drugs such as doxorubicin, temozolomide and paclitaxel [67]. The proapoptotic effects observed in the majority of the discussed studies might be explained when taking a closer look at some factors involved in apoptotic pathways. NF-κB-luciferase transfected PC-3 cells were treated with a combination of tumor necrosis factor α (TNF-α) and tomato extract. The expression of proapoptotic genes, FAS and caspase 9 (CASP9), were higher, although the expression of hypoxia-inducible factor 1-α (HIF1α) was also increased when compared to the TNF-α control [74]. Another important endpoint for such cellular trials is colony formation, being proportional to the aggressiveness of the cells. Usually, PC-3 cells create the largest number of colonies, while DU145 produce fewer. LC has been shown to reduce the colony formation in both cell lines. PC-3 created 110 (20 μM) and 59 (50 μM) in comparison to 180 colonies (control). DU145 produced 76 (20 μM) and 35 (50 μM), while untreated cells formed 115 colonies. Treatment with 20 μM and 50 μM TL reduced the colony number to 135 and 83 in PC-3, whereas in DU145 colony numbers were 90 and 57, respectively [69]. As the vast majority of the effects of LC are suggested to occur via intracellular pathways, its ability to penetrate through the cellular membrane is a highly important issue. PrEC, LNCaP, C4-2, DU145, and PC-3 cells were treated with 1μM and 3 μM LC for 24 h. The highest concentration was observed in PrEC (150 and 250 pmol/10^6^ cells, respectively). LNCaP accumulated 75 and 200 pmol/10^6^ cells, C4-2: 10 and 50 pmol/10^6^ cells, DU145: 20 and 80 pmol/10^6^ cells, PC-3: 60 and 100 pmol/10^6^ cells, respectively. The primary conclusion is that PC cells tend to accumulate less LC than healthy prostatic tissue, perhaps due to altered expression of transporters [104]. Additionally, the weaker the differentiation grade, the lower the intracellular concentrations of LC [62]. Surprisingly, 20 μM of AL caused an accumulation of 46 pmol/10^6^ cells LC in PC-3 cells, while 50 μM showed 69 pmol/10^6^ cells. The same concentration of TL caused an accumulation of 40 and 58 pmol/10^6^ cells, respectively [69], also pointing out that cellular accumulation of LC does not depend linearly on its surrounding concentration and thus does not define its potency to exert any intracellular effects, which is in line with human supplementation trials [55].

The most dangerous feature of cancer is not only its ability to grow locally but also metastasize. Lowering cellular ability to spread to distant locations is therefore as paramount as reducing cellular proliferation. Few investigations including LC did focus on this issue. Treatment with 2.3 µM LC was shown to remarkably inhibit adhesion of PC-3, DU145 and PNT2 (immortalized normal prostate cell line). This effect was most pronounced for DU145 cells, while PNT2 had migrated faster than the cancer cells. When administering half the dose, LC reduced the attachment of PC-3 and PNT2 but not DU145 cells [75]. Some proteins known to be responsible for controlling adhesion and migration of PC (namely intercellular adherence molecule 1 (ICAM1) and MMP9) were downregulated in PC-3 cell lines treated with tomato extract [74]. These results point out the ability of LC to diminish to some degree PC metastatic abilities.

#### 5.1.3. Lycopene—Hydroxymethylglutaryl-CoA Reductase (HMG-CoAR), PPARγ, LXRα and Adenosine Triphosphate-Binding Cassette Transporter Subfamily A Member 1 (ABCA1) Pathway

Steroid hormones play a crucial role in the pathogenesis of PC. The pathway of synthesis of their endogenous precursor—cholesterol—is a potent target for many drugs directed against PC. Indeed, it was shown that LC reduced concentrations of cholesterol in LNCaP cells in a dose-dependent manner for a wide range of concentrations (from 2.5 μM to 10 μM), causing a 56% decrease in cholesterol levels when administered at the maximal stated dose. Yet, 2.5 μM was the lowest concentration of LC that was able to inhibit the activity of HMG-CoAR, the enzyme controlling cholesterol production [76]. This was confirmed by another study with the same concentrations of LC (for LNCaP and DU145 cells). Surprisingly, cholesterol in the cellular medium was increased, suggesting some shift of this lipid instead of its absolute depletion. All the effects weakened with time but did not diminish totally after days. The measurement of the apolipoprotein AI (ApoAI) protein and the encoding mRNA showed their upregulation. Similarly, HMG-CoAR was also downregulated in the PC-3 cell line [64,65]. It is worth noting that the inhibition of cholesterol synthesis targets also growth-controlling pathways through impairing the activity of small G proteins (such as Ras), which demand farnesylation for proper action. Cell lines with mutated, less-stable Ras proteins were 2–3 times more prone to the LC treatment than those without Ras mutation [77].

Further pathways engaged in cholesterol metabolism have also been investigated. DU145 cells treated with 10 µM LC for 12 h, 24 h and 48 h showed that the level of proteins and mRNAs encoding PPARγ, LXRα and ABCA1 were increased, with the strongest effects observed after 24 h of incubation [64]. The same protocol was repeated for LNCaP and concomitant results were observed [65]. In subsequent experiments, DU145, PC-3 and LNCaP cells were treated with T0901317, an LXR agonist. The level of LXRα was unchanged, but the expression of ABCA1 increased. A selective antagonist of PPARγ (GW9662) or LXRα (GGPP) abolished the effect of LC, changing the expression of these proteins in LNCaP cells below the levels expressed in the control cells.

#### 5.1.4. Lycopene—ROS, NF-κB and Akt

Incubation with LC significantly reduced the level of ROS in LNCaP cells. After a 3 h incubation period, the amount of ROS measured by a fluorometric assay was significantly reduced, in a dose-dependent manner, starting from 2.5 μM. ROS activated NF-κB, which modulates the synthesis of many proteins involved in cell cycle and apoptosis regulation, being the central coordinator of the inflammatory state. In tumor cells, the level of this protein is often increased, and its reduction, therefore, seems to be desirable in the process of oncological treatment. LC inhibited the activity of this transcriptional factor, likely due to its ability to diminish ROS [68,77]. One day of treatment with LC reduced levels of cyclin D1, B-cell lymphoma 2 (Bcl-2) and Bcl-XD in LNCaP cells. All these proteins are effectors of NF-κB. Additionally, the level of p53, p27, p21 and Bcl-2-associated X protein (Bax) increased, concomitant with inhibitory effects on cellular division. Treatment with mevalonate abolished these effects, indicating that the HMG-CoAR pathway could be more important for reducing NF-κB than ROS [77].

To confirm that the aforementioned effects were mediated by NF-κB, a luciferase reporter assay was developed [74]. NF-κB-luciferase positive cells (PC3-κB-luc cells) were treated with different concentrations of tomato extract alone or with TNF-α. The highest used LC concentration, 8.6 μM (without TNF-α), caused a 78% increase in NF-κB activity, compared to the negative control (but it was less than 10% of the TNF-α positive control). In PC-3 cells, a mixture of tomato extract and TNF-α inhibited the NF-κB activity in a dose-dependent manner, starting from 3.5 μM LC, compared to the TNF-α control. The inhibition was significant for 5.8 μM and 8.6 μM (53.3% and 59.2% respectively). In the next step of the experiment, the PC3-κB-luc cells were injected into male NMRI nude mice. These mice were fed a control diet or one containing 10% tomato paste. No significant difference between the control group and the treated group was observed regarding tumor growth. However, the level of NF-κB activity was lower in tomato-fed mice compared to the control. Three weeks after injection, a 27.3% reduction was observed, and at 5 weeks the reduction was still 14.3%. In this model, the expression of the transforming growth factor-beta 1 (TGF-β1) gene was reduced, while the levels of TNF-α and ICAM 1 genes were increased by tomato paste. In the next step, the specific mRNA in PC-3 xenografts was analyzed. The NF-κB subunit 2, signal transducer and activator of transcription 3 (STAT3), STAT6, suppressor of cytokine signaling 2 (SOCS2), TGF-β signaling repressor (SKI), which are all able to interrupt inflammatory signaling, were upregulated. Furthermore, the interleukin-18 (IL-18) and endothelin converting enzyme 1 (ECE1) gene activities were reduced [74]. Thus, there was a noticeable anti-inflammatory shift in the behavior of the cells. Despite this demonstration of the implicated NF-κB pathway, the mechanism by which LC interacts with NF-κB remained unclear. To investigate this question, the rate of phosphorylated inhibitor of kappa B (IκB) was determined in another study. Treating PC-3 cells with 1.25–5.00 µM LC for 20 h resulted in a 30–40% reduction in p-IκB [72]. A similar effect was obtained after 30 min of TNF-α treatment, where dephosphorylated IκBα inhibited the NF-κB signaling pathway. For confirmation, PC-3 cells were treated with 5 μM LC for 2 h. After this time, TNF-α was added to the cells and incubated for 1–5 h. LC inhibited p65 nuclear translocation by at least ∼25% between 2 to 5 h. The strongest effect was observed after 2 h and was 60%. The p65 nuclear/cytoplasmic ratio was ∼2.5 × lower compared to the control. Interestingly, there is evidence that it is not LC, but rather its metabolic derivatives, which are responsible for such effects [76]. Similarly, 72 h of LC incubation (0.5–5.0 μM) inhibited the transcription activity of TNF-α, with reductions of 20–50% in PC-3 cells, starting at a concentration of 1.25 μM [72].

Similar as to inflammation and NF-κB, Akt kinase is a pivotal junction in which signals from numerous growth factor receptors are coming together and are further transduced. These growth-stimulating signals might be generated by a phosphorylated form of the Akt kinase. Consecutively, it was hypothesized that LC might switch the Akt to an unphosphorylated inactive state. LNCaP and DU145 cells treated with 0.5, 1 or 10 µM LC for 24 h showed that levels of phosphorylated Akt decreased, without changes in total Akt protein level. Inhibitory effects of Akt activation for the highest concentration were more severe in DU145 (60% reduction) than in LNCaP (20% reduction) cells. Most likely, this could be explained by loss of phosphatase and tensin homolog (PTEN) (responsible for dephosphorylation of Akt) in LNCaP cells [63,78]. More recent experiments studied levels of Akt2 and the activity of phosphatidylinositol 3-kinase (PI3K)/Akt signaling pathways. PC-3 cells treated with 10, 25 and 50 µM LC incubated for 48 h revealed that the Akt2 level was reduced by 19, 42 and 67%, respectively. Similar results (18, 42 and 52%) were achieved for Akt.

The miR-let-7f is a miRNA, which targets Akt2 mRNA and thus inhibits cellular proliferation. Applied LC concentrations increased the expression of miR-let-7f by 74, 131 and 188%, respectively. Transfection with miR-let-7f alone significantly decreased LNCaP cell proliferation. These results suggest that induction of miR-let-7f is another mechanism by which LC inhibits cell proliferation [80]. Finally, not only Akt but also other proteins showed a lower phosphorylation status after LC treatment, including p-JNK, p-Erk1/2 and p-p38 [77].

#### 5.1.5. Lycopene—Proapoptotic and Antiapoptotic Proteins and Cell Cycle

In some slowly developing tumors, immortalization through the inactivation of apoptotic pathways is a basic mechanism for growth. It was earlier shown that 5 and 10 µM LC treatment increased the activity of Bax and cytokeratin 18 (CK18) and simultaneously reduced the activity of Bcl-2 after 96 h of incubation [68]. The same results were observed in an in vivo study. Six to seven-week-old BALB/c nude mice were fed with low (9 mg/kg) or high LC doses (18 mg/kg body weight) for 2 weeks and were then injected with PC-3 cells. The mice were sacrificed after seven weeks. Treatment with LC (18 mg/kg) reduced the tumor volume (51 ± 8 mm^3^ vs. 248 ± 29 mm^3^ in the control group). The level of Bcl-2 decreased and Bax increased in these tumors [79]. Tomato-based food extracts (described in Section 5.1.2) showed similar changes, but were still different depending on the particular product. LC from tomato paste increased Bax 52.9-fold, from ketchup and tomato extract 2.2-fold. Tomato sauce reached only a 1.2-fold increase. Ketchup and tomato extract increased tumor protein 53 (TP53) gene expression 5.7-fold, tomato paste 16.8-fold and tomato sausage only 1.9-fold. The level of Bcl-2 was decreased 1.2-fold with LC from tomato paste treatment and 1.0-fold with LC from tomato extract, while tomato sauce reached no significant result [70]. This result is concomitant with a 35% inhibition of Bcl-2 expression measured in microarrays, with similar effects for the Bcl2l1 gene [67]. One of the studies investigated the effect of LC on insulin-like growth factor 1 (IGF-1). Cells taken from patients with a Gleason score (GS) of 6 were treated with 1 µM, 2 µM and 4 µM of LC. No change was observed in the group treated with the 1 µM solution. IGF-1 levels of 0.112 ng/mL were observed for the group treated with 2 µM LC, followed by a change in IGF-1 levels to 0.760 ng/mL after 48 h and 0.690 ng/mL after 72 h. Treating cells with 4 µM increased IGF-1 levels to 0.785 ng/mL and then reduced them to 0.680 and 0.515 ng/mL after 48 and 72 h, respectively. However, the concentration in the control sample was only 0.112 ng/mL after 24 h, 0.113 ng/mL after 48 h and 0.1125 ng/mL after 48 h, indicating an increase in the concentration of this growth factor during the action of LC [80].

To definitively conclude whether changes in apoptotic proteins, ROS or growth kinases are real effectors of LC in PC, detailed investigations on the cell cycle were carried out. A 24 h treatment with LC in LNCaP cells resulted in an increase in the percentage of cells in the G0/G1 phase, with reductions in the S-phase. The presence of a distinct sub-G1 peak (subdiploid DNA content) was noticed, suggesting that some cells had entered the state of apoptosis. Increased activity of CASP3 was also found [77]. On the other hand, treating cells with paste and tomato extract (500–5000 µg/mL) for 96 h decreased the number of cells in the G0/G1 and G2/M phases. However, tomato sauce and ketchup administered at the same concentrations hampered the percentage of cells in the G0/G1 phase, with a simultaneous increase in the number in the S and G2/M phases [73].

Similarly, treatment with a combination of LC and docetaxel significantly increased the number of DU145 cells in the pre-G1 phase, compared to treatment with docetaxel alone [63]. The percentage of DU145 cells in the G0/G1 phase also increased, whereas in the G2/M phase the reduction was noted after 48 h treatment with LC, although after 96 h a higher percentage of cells in S and G2/M phases was noticed with a decrease in the G0/G1 phase. In PC-3 cells, an increase in the S and G2/M phases and a reduction in the G0/G1 phase were observed for both durations [68]. These findings were repeated in another study [67]. Additionally, combinations of LC and different drugs were shown to prevent cells from entering the later stages of the cell cycle. Doxorubicin treatment of PC-3 cells showed 88% debris (subG0/G1), however, in combination with 25 μM of LC, this increased to 92%. The subG0/G1 fraction is also referred to as apoptotic cells. Cells treated with docetaxel showed 26% apoptotic cells; together with LC, this increased to 33%. PC-3 cells treated with LC and 15dPGJ_2_ or ciglitazone showed a 2-fold increase in the number of apoptotic cells, though no change for pioglitazone was observed [67]. PC-3 cells after 24 h of incubation with 20 μM AL were at 69% in the G0/G1 phase, 10% in the G2/M phase, and 7% in the S phase (control: 24% in G0/G1 phase, 24% in the G2/M phase, 29% in the S phase). A lower effect was observed in the TL treatment group. After 20 μM TL treatment, 60% of cells were in the G0/G1 phase, 11% in the G2/M phase and 7% in the S phase. These results suggest that LC caused PC-3 cell accumulation in the G0/G1 phase and apoptosis. All of these changes in the cell cycle might be hypothesized to follow changes in levels of some proteins engaged in cell cycle regulation. Namely, 25 µM of LC inhibited cyclin-dependent kinase 7 (Cdk7) and 9 by 25% and 100%, respectively. Genes for proteins important for cell proliferation and survival, such as epidermal growth factor receptor (EGFR) and TGF-β2 were also downregulated (by about 60% and 83% respectively) [69]. Analysis of these outcomes confirms that LC prevented the progression in the cell cycle of PC cells, thus fostering apoptosis.

#### 5.1.6. Lycopene—Gene Methylation, Glutathione S-Transferase P1 (GSTP1), IGF-1

GSTP1 is among the genes most silenced by hypermethylation in PC (>90% of cases), which happens also in the LNCaP cell line. LNCaP cells treated with 1 μM, 2 μM, and 4 μM of LC were incubated for 7 days. LC was taken up in amounts of 42, 61, and 78 pmol per million cells for 1 μM, 2 μM, and 4 μM, respectively. The expression of GSTP1 and DNA methylation of the GSTP1 promoter did not change significantly, suggesting that LC did not have an influence on the demethylation of the GSTP1 gene promoter [81].

IGF-1 is known to be responsible for a part of insulins’ effect in the entire body. Additionally, the receptor for IGF-1 belongs to the family of tyrosine kinase-associated receptors. Thus, its effects on the growth of PC have been investigated. All PC cell lines presented the same dependency—the higher the expression of the insulin-like growth factor-I receptor (IGF-IR), the more visible the impact of LC on their growth. Estimated IC_50_ values inversely depended on the IGF-IR level: DU145—5.1 µM; PC-3—15 µM; 22Rv1—16 µM; LNCaP—36 µM and LAP-4—50 µM. The corresponding levels of IGF-IR were 9.3; 4.1; 1.0; 2.0 and 0.8 (relative units, the baseline 1.0 represents IGF-IR expression in 22Rv1). Experiments that compared parental LNCaP and LNCaP, stably expressing high levels of IGF-IR (LNCaP/IGF-IR) showed that LNCaP/IGF-IR were 400-fold more susceptible to LC treatment. The IC_50_ value for LNCaP/IGF-IR reached 0.08 µM. It was shown that IGF-IR is possibly another effector of LC. To confirm this, PPP (a selective IGF-IR kinase inhibitor) was used. LNCaP/IGF-IR growth was 7-fold more inhibited by PPP than parental LNCaP growth. DU145 cells incubated for 2 h with LC or PPP presented IGF-I-induced inhibition of IGF-IR phosphorylation, without increased IGF-IR levels. Therefore, LC interferes with the activation of IGF-IR or IGF-IR kinase in response to IGF-I. In addition, LNCaP/IGF-IR cells had higher levels of active Akt and antiapoptotic protein survival compared to the parental LNCaP cell line. Twenty-four hours of incubation of DU145 cells with LC resulted in a dose-dependent enhancement of IGF-BP3 (the negative regulator of IGF-1) protein expression and secretion [63]. Additionally, in the PC-3 cell line, IGF-1R was downregulated by LC (with a 50% inhibition of expression for 25 µM LC) [67]. These results strongly support the theory that blocking the IGF-1 pathway is a way of LC action and probably would be also engaged in inhibiting native PC, although this remains to be shown.

#### 5.1.7. Lycopene—BCO1 and BCO2

BCO2 is present in the healthy human prostate, however, it is somewhat weakly expressed in PC. The level of BCO2 was measured in different prostate cell lines. The highest concentrations were detected in PrEC cells, and it also was high in androgen-sensitive LNCaP and C4-2 cells [62]. The lowest expression was observed in DU145 cells. Some data suggest that the BCO2 gene might be transcriptionally regulated by epigenetic mechanisms, thus cell lines were treated with 5-aza-2dC (the methyltransferase inhibitor) to investigate this. The BCO2 gene activity significantly increased in PC lines (LNCaP, PC-3, C4-2, DU145), but not in PrEC cells, confirming that the methylation levels of BCO2 promoter in this line were initially low. In the next step, LNCaP and DU145 cells were treated with 1 μM LC for 24 h. In LNCaP cells, the level of BCO2 increased after LC treatment (with accompanying reduction in proliferation), although in DU145, a model of more aggressive PC, it was not changed. This suggests that BCO2 levels are decreasing during PC progression. The final confirmation of the role of BCO in PC was achieved by transfection with cytomegalovirus (CMV) vectors (pCMV-BCO1, pCMV-BCO2). The control was created by transfecting an empty vector (pcDNA3). Transfected cells were treated with or without 1 μM of LC for 24 h. In LNCaP and DU145 cells with overexpressed BCO2, LC significantly inhibited cell proliferation. However, this effect was not observed in cell lines overexpressing BCO1 [62]. This suggests that BCO2 inhibits tumor growth in a way different from its own direct enzymatic activity. To confirm that BCO2 was silenced through mutation of its catalytic domain (BCO2-mt), colony formation was inhibited by LC in DU145 cells transfected with either BCO2 or BCO2-mt, confirming the hypothesis. In the next step, the authors examined the effect of BCO2 and LC on NF-κB. DU145 cells were transfected with an NF-κB luciferase reporter construct and one of three vectors used before (null-vector, BCO2 or BCO2-mt). After that, the cells were treated with LC for 24 h. LC alone did not induce any changes. Transfection with BCO2 and BCO2-mt significantly reduced the level of NF-κB, however, no further changes were triggered by LC. BCO2 and BCO2-mt also abolished the effect of TNF-α on the stimulation of the NF-κB activity. The authors also showed that BCO2 reduced nuclear translocation and DNA binding of the NF-κB p65 subunit [62]. This study revealed that LC modulates the carotenoid metabolizing enzyme BCO2, giving some insights into the observed interactions among different carotenoids. Surprisingly, NF-κB was not affected by LC at all in this study, which is inconsistent with previously discussed results. However, in addition to using DU145 cells (instead of PC-3 and LNCaP cells), LC was used at lower concentrations and thus its threshold level for inhibiting NF-κB might have not been reached.

### 5.2. Lycopene—Mice Models

Biological interactions in living organisms are far more complex than the ones present in vitro. After the acquisition of data in cellular models, in the following, we summarize results from animal models.

As the IGF-1 pathway was proposed to be one of the most potent targets of LC (see previous sections), it was further investigated in the TRAMP mouse model. Rodents were fed 28 mg (per kg of feed) LC per day in form of tomato powder (TP) or LC beadlets (LB) for 20 weeks. The authors evaluated their prostate histopathology, serum levels of IGF-I and insulin-like growth factor binding protein 3 (IGF-BP3). Mice fed LB demonstrated a significantly reduced incidence of PC compared to the control group (60% vs. 95%; *p* = 0.0197). About 30% of the mice in this group developed BPH, which was not observed in the remaining population. Changes in the incidence of PC in the TP group were not statistically significant. Serum levels of IGF-I and IGF-BP3 were unchanged and no difference among groups was observed. In the next step, wild-type (WT) mice were fed in the same way. The levels of serum LC were the same in LB and TP mice, while being undetectable in controls. However, the ratio of serum 5-cis to all-trans-LC was higher in LB mice, indicating that LC beadlets efficiently raised the bioavailability of this compound [82].

Another study investigated TRAMP mice at four weeks of age randomly assigned to one of the three groups: placebo beadlets, tomato powder (providing 384 mg LC/kg diet) and LC beadlets (providing 462 mg LC/kg diet). The dosages of LC used were about 15-times higher than in previous studies. WT mice of the same age were assigned following a similar pattern: LC beadlets providing 20 mg LC/kg, tomato diets providing 40 mg LC/kg diet and a control diet. After four weeks, all mice groups were randomized to various surgical procedures: a sham (superficial incision only) surgery, castration and castration followed by testosterone repletion (2.5 mg testosterone propionate/kg/day). Testosterone was administered for seven days after castration. Twelve days after surgery, each mouse was sacrificed. WT and TRAMP mice fed with LC beadlets or tomato powder had the same concentration of LC in their plasma. Castrated mice in both dietary groups had lower plasma LC concentrations than the sham surgery group. The diet containing tomato and LC had an impact on 30 genes (the activity of 26 was decreased and 4 increased). LC and tomato treatment reduced the expression of genes involved in androgen metabolism and signaling. Genes encoding the isoforms of 5α-reductase (Srd5a1, Srd5a2) were downregulated in tomato and LC-fed mice. Tomato-fed mice had lower levels of two AR co-regulators—Pxn and Srebf1. Genes inhibited by these dietary modifications included also those active in neuroendocrine differentiation and stem cell-related ones (Ngfr, Aldh1a1). Ki-67 protein (a marker of proliferation) expression did not change [18]. This study showed that LC interfered with androgen signaling directly (through diminishing Srd5a expression), which could be a promising way of preventing PC growth and development.

In search for some combinatory supplement methods of prevention and therapy, Lady transgenic mice (12T-10) received vitamin E (α-tocopherol succinate—800 IU), selenium (seleno-DL methionine—200 μg) and LC (50 mg) for 42 weeks. In control mice without supplementation, 90% developed PC during that period of time. However, in supplemented mice, only 15% developed PC. One of the most upregulated peptides in their sera was platelet factor-4 (PF-4), an inhibitor of angiogenesis. Therefore, prostates were immunoassayed for PF-4 and α_2_β-integrin (cluster of differentiation 41 (CD41)—a carrier of PF-4 in vivo). Prostates from supplemented mice increased significantly in the expression of PF-4 in their vessels, in contrast to mice fed with a standard diet. Similar results were obtained for CD41, the majority of supplemented mice exhibited this protein on the platelet surface in their prostatic vessels, but none did so in the control group. The authors suggested that PF-4 blocked angiogenesis at an early stage of tumorigenesis and the production of this protein by megakaryocytes was potentiated by a combination of supplements [83].

It was important to determine whether LC preserved the ability to abolish androgen insensitive DU145 cells when administering them to an in vivo model (as this line was generally most prone to the LC treatment in vitro). DU145 cells (1 × 10^6^) were injected into the right flank of NCR-nu/nu (nude) mice. When the tumors reached volumes of 200 mm^3^, the mice were divided into several groups. The ones treated with higher doses of docetaxel (10 mg/kg per week) or a combination of an LC supplement with a lower (5 mg/kg per week) or higher (10 mg/kg per week) dose of docetaxel group developed PC significantly slower than the control group, or LC alone or low-dose docetaxel. The LC supplement in combination with the lower dose of docetaxel had the same efficacy in prolonging the life of mice than the higher dose of docetaxel. In histologic analysis, the DU145 xenograft-bearing mice treated with LC and docetaxel showed significant changes in tissues and cellular morphology compared with other treatments. Low cellular density and multinucleated cells with condensed chromatin staining and pyknosis, indicating mitotic catastrophe and apoptosis, were observed. Next, these tumor xenograft tissue sections were examined by TUNEL and immunohistochemistry for Ki-67. LC with docetaxel increased the level of apoptotic cells by 98% or 392% compared to docetaxel or LC alone, respectively (which was followed by reduced survival level). There was no statistically significant difference for the Ki-67 test [63]. The results indicated that LC could constitute a potent agent for combination therapy.

### 5.3. Lycopene—Mechanistic Studies in Humans

The actions of carotenoids on the human body can be confirmed only through direct evidence by carotenoid effects in human studies. “The Molecular Effects of Nutritional Supplement” (MENS) trial studied effects of supplementation with 30 mg/day of LC for 3 months in 84 men with a low risk of PC. The point of concern in this study was the hypothetic interaction of IGF-1 and its receptor (IGF-IR) in healthy prostatic tissue (not affected by tumor growth). As the measured entity mRNA was chosen. This study resulted in almost no effect of LC on IGF-1 and IGF-1R mRNA. ΔCT values for the placebo and intervention group were similar (0.18; *p* = 0.93). Additionally, for IGF-1R, no changes were observed (*p* = 0.53). After measuring PSA concentrations, no difference was reported [105]. Limited conclusions can be drawn from this study, as only healthy prostatic tissue was investigated. As the higher expression of IGF-1 increased the susceptibility to LC in PC cell lines, it could be hypothesized that similar effects would be observed in native PC. According to the presented study, this could not be stated for certain—as it is unclear whether no change in mRNA is equal to no change in protein activity or expression, due to the widely reported impact of carotenoids on the translational process and PTMs of proteins. Additionally, the study did not adjust for age or other important factors.

The samples were used again in another study, analyzing basic molecular pathways in normal prostate tissue after a 3-month supplementation of 30 mg/day of LC. Following complementary DNA (cDNA) microarrays, no changes for any genes were significant. The authors then decided to analyze numerous molecular pathways. A more than 1.5-fold increase was observed for Nrf2-mediated oxidative stress response, apoptosis and ceramide signaling, glutamate metabolism, glutathione metabolism and PXR/RXR activation. The most pronounced difference was Nrf2-mediated oxidative stress relief [106] showing again the importance of this pathway for carotenoid action.

The last study aiming to assess the molecular actions of LC in humans was performed in 2015. A group of 10 men with high-grade prostatic intraepithelial neoplasia (HGPIN) and/or atypical small acinar proliferation (ASAP) were treated with 35 mg of LC, 55 μg of selenium and 600 mg of green tea polyphenols daily or with placebo for 1 month (phase I). Then, after periodic evaluation, 50 new participants were enrolled, all 60 participants were again randomized into equal groups (30 men in each, preserving the same schedule of allocation as in phase I) and the study continued for 6 months (phase II). At the end, each participant underwent 12-core prostate biopsies. Phase I was intended to obtain data on the concentration of LC in sera, stability, and potential adverse reactions, whereas in phase II the presence (and possibly grade) of PC or HGPIN/ASAP was of main interest. From eight patients, prostatic tissue (from tumor affected sites) was taken for miRNA expression profiling. In this study, a high medium concentration of LC in the serum of participants was achieved (14.5 µM). At the end of the study, ten PC were found in the supplemented group and three in the placebo group (*p* = 0.053). However, at follow-up, three more PC occurred in the placebo group (one of GS 9, while no other case exceeded GS 7) and none in the active arm of the study. This reduces the clarity of the results, and it is apparent that such a study is vastly underpowered and rather has a pilot character.

The molecular analysis in the supplementation group revealed a significant increase in 39 miRNAs (the authors’ interest was not direct concentrations, but changes between initial biopsy and re-biopsy). Overexpressed miRNAs were let-7f-5p, miR-100-5p, miR-130a-3p, miR-23a-3p and were reported to be associated with the presence of cancer in general, while miR-15a-5p, miR-26b-5p, let-7i-5p, let-7d-5p, miR-16-5p, miR-199a-5p, miR-214-3p, miR-15a-5p, miR-29b-3p, miR-30e-5p, and miR-34a-5p were often found in PC directly. In contrast, the strongest reduction was observed in miR-494 expression, which was associated with suppressed tumor growth. These results support the fact that patients with PC showed increases only in miR-16-5p and miR-100-5 in regard to miRNAs associated with unfavorable effects. The remaining increases in PC miRNAs were: miR-193b-3p, miR-92a-3p, miR-10b-5p, miR-103a-3p and miR-125b-5p [58]. The authors concluded that such supplementation could act rather as a “chemopromotion” than chemoprevention of PC. However, when taking a closer look at the two miRNAs that increased simultaneously in the supplementary group and PC, there is some inconsistency. Data from the OncomiRDB database suggest that both miR-16-5p and miR-100-5 inhibited tumor growth and reduced cell proliferation [107]. In addition, one of the in vitro studies (presented above) was proving that miR-let-7f, upregulated by LC, acted as a negative regulator of proliferation, instead of being merely a marker of tumorigenesis [77]. Conclusions based solely on miRNA should be drawn with care, as their biology is complex and new aspects are still discovered.

Finally, as a rather classical marker of PC, studies have also investigated the effect of LC on PSA concentrations. Of 41 patients who were diagnosed with PC before, 37 were supplemented with 10 mg of LC per day. A decrease in PSA level was observed in 26 out of 37 individuals. In eight patients, the PSA level increased non-significantly after 1-month follow-up [62]. The results are only partially comparable to in vitro results, demonstrating that LNCaP cells treated with 2.5 μM LC did not change PSA secretion and ARE gene activity [71].

In one of the studies, the effect of SNPs of the following genes was determined: BCO1, ABCA1, ABCB1, scavenger receptor class B type 1 (SCARB1), an intergenic SOD2, microsomal triglyceride transfer protein (MTTP), elongation of very long-chain fatty acids protein 2 (ELOVL2), apolipoprotein B-48 and mitochondrial-associated SNP. These were compared to the concentrations of LC, BC, phytoene, and phytofluene in the blood, and prostate tissue after prostatectomy. A total of 47 PC patients received 0, 1, or 2 cans of tomato-soy juice/day (163 mL/can; 20.6 mg lycopene and 1.2 mg β-carotene/can) for 24 ± 0.7 d before prostatectomy. The results showed that only the polymorphisms of the BCO1 gene affected the concentration of LC and BC. Polymorphisms of this gene in two loci (rs12934922, rs6564851) affected the concentration of LC in the plasma, depending on the amount of LC consumed. The polymorphism in the rs12934922 locus had an influence on the concentration of LC in prostate tissue. In the case of BC, statistically significant changes in plasma concentrations occurred related to the variants of rs12934922 locus, while in the cases of prostate tissue loci rs12934922 and rs7501331 had an influence. This study suggested that different gene variants are responsible for the level of carotenes and thus their potential impact on the prostate and the entire human body, which may be of great importance when regarding the prevention and treatment of PC [108].

One more aspect investigated in the context of LC and PC is metabolomic studies. The ProDiet randomized controlled trial (RCT) [109] investigated the effects of a 3-month LC and green tea supplementation. A total of 133 men between 50 and 69 years of age with elevated PSA levels and a negative prostate biopsy result were divided into three groups. The first received LC tablets (n = 40), an LC-rich diet was recommended for the second one (n = 43) and the third received a placebo. Unfortunately, the exact doses of LC taken were not provided for all arms, impeding the outcome interpretation. It was shown that LC supplementation lowered plasma concentrations of valine, pyruvate, diacylglycerol, and docosahexaenoic acid, while increasing in acetate concentration. High pyruvate was associated with an increased risk of PC development. Thus, LC appeared to protect against PC.

### 5.4. Lycopene—Other Investigations

One study assessed the effect of LC on PC in an intriguing way. Thirty healthy male vegetarians, 50 to 70 years of age with a normal biochemical blood profile and without any chronic diseases or taking medication participated. The volunteers were randomly assigned into two groups. Group 1 received a daily supplement (200 g/d for 1 week) of yellow tomato paste (YT), while group 2 consumed the same amount of red tomato paste (RT). Next, after a 2-week washout period, group 1 received the RT and group 2 the YT. After the next washout period (2 weeks), group 1 received a daily capsule providing LC at the same concentration as the RT (16 mg/d, LYS) while group 2 consumed a placebo capsule daily for 1 week. After completion, blood and urine samples were taken. The levels of PSA and IGF-1 were the same in both groups. The level of LC was much higher in the RT than in the YT group. In fact, serum LC in the YT did not change when compared to the baseline. BC concentration also increased in both groups, but the effect was more pronounced in the RT group. Plasma antioxidant capacity, PSA and IGF-1 level, urinary F_2_-isoprostanes were similar in all phases. In the second step, LNCaP cells were incubated for 48 h with the sera of volunteers from all groups. After that, the authors measured the mRNA levels of 25 genes in white blood cells, involved in prostate carcinogenesis. The mRNA level of eight genes, i.e., α-reductase-1, estrogen receptor-1 (ER-1), E-selectin, MMP-9, vascular cell adhesion molecule (VCAM), cyclooxygenase-2 (COX-2), IL-6 and IL-1α—were unchanged. After incubation with serum from the red tomato sera (RTS) group the amount of cyclin D1, p53, and Nrf-2 decreased, though the Bax: Bcl-2 ratio and IGFBP-3 level did not change. There were no statistically significant results after incubation with yellow tomato sera (YTS). In contrast, the amount of IGFBP-3, c-Fos and uPAR increased after incubation of serum from the LYS group. A reduced proliferation was found after treatment with 5 μM LC, but this concentration was not achieved in any of the sera (60, 30, and 20 nM for RTS, YTS, and placebo sera (PoS), respectively) [110].

### 5.5. All-Trans-Retinoic Acid, Retinol and Vitamin A

ATRA targets many different NRs, the main ones being RARs and RXRs. However, ATRA may act via various pathways, which are not associated with these receptors. In cell lines treated with ATRA, increased cytotoxicity was observed in a dose- and time-dependent manner. This effect was more visible in androgen-insensitive cell lines, especially in the DU145 cell line [84]. ATRA can trigger many effects, inspiring research on the influence of its conjugates on amino acids. Statistically significant results were found only for conjugates of ATRA with leucine and β-alanine. A decrease in the number of LNCaP cells was observed in a concentration-dependent manner when exposed to the maximum concentration used (10 μM). To control whether ATRA and its conjugates decreased cell proliferation through a RARα-dependent way, LNCaP cells were treated with the RARα selective antagonist Ro415253. Ro415253 inhibited the effects of ATRA, but the decrease in the number of LNCaP cells caused by conjugates with leucine and β-alanine was not totally counterbalanced. It was suggested that the mechanisms of action of these conjugates were not the same as for ATRA [85].

Research on carotenoids and metabolites, including ATRA, is not limited to isolated substances. The trend in anticancer therapy is polytherapy, which has also motivated the exploration of the potential synergy between ATRA and zoledronic acid, a drug from the group of long-acting bisphosphonates. PC-3 and DU145 cells were treated with different concentrations of ATRA and zoledronic acid alone or in combination for 24 h, 48 h and 72 h. A strong synergistic toxicity was detected at 72 h. ATRA alone in the concentration of 80 nM decreased PC-3 cell viability by 39%, 40 μM zoledronic acid reduced it by 28%, whereas their combination resulted in a 75% decrease. The viability of DU145 cells treated with 40 nM ATRA and 20 μM zoledronic acid was decreased by 23% and 24%, respectively, while combined they reduced viability by 60%. Additionally, synergistic effects of these compounds were seen on the induction of apoptosis, increasing DNA fragmentation in both cell lines (dose-dependent manner).

A significant increase in the activities of CASP3, seven enzymes were detected in PC-3 and DU145 cells in response to ATRA and zoledronic acid. Again, their separate effects were surpassed by their combination, which triggered an 8.2-fold increase in the level of CASPs. The mRNA level of tumor necrosis factor receptor superfamily (TNFRSF) genes also increased. However, expression levels of antiapoptotic gene family members, namely baculoviral IAP repeat-containing protein 2 (BIRC2), BIRC5, myeloid cell leukemia 1 (MCL-1) and lymphotoxin beta receptor (LTβR), decreased after exposure to both substances, alone and combined. The same results were obtained by oligoarray and real-time PCR. The levels of proapoptotic proteins, Bad, Bax, Fas, and Fas-associated protein with death domain (FADD), smac/diablo increased, but the amount of Bcl-2, p53 and BIRC5 proteins were lower by a combined treatment of ATRA and zoledronic acid. Therefore, treatment with ATRA and zoledronic acid seems a promising therapeutic option [84].

The HOXB13 gene is a member of a large homeobox superfamily and a TF, active in axial regions of the body during embryonic development. It also plays an important role in inducing terminal cell differentiation in the prostate. The HOXB13 gene is inactivated in androgen-insensitive PC cells. This research examined the effects of treatment with ATRA on the methylation of the HOXB13 gene and cell cycle arrest induced by it. DU145 cells were treated with different concentrations of ATRA (20 to 120 µM) for 24, 48 and 72 h. ATRA induced cell growth arrest in DU145 cells, with strongest effects at 72 h. The level of expressed HOXB13 mRNA and protein significantly increased after ATRA treatment. Silencing the HOXB13 gene by siRNA reversed the antiproliferative effects caused by ATRA. In various cancers, including PC, the HOXB13 promoter is hypermethylated, but this condition is more severe in DU145 than in LNCaP cells. Using specific siRNA demonstrated that the DNA methyltransferase 3b (DNMT3b), an enzyme that plays an important role in the methylation of many cancer genes, also involved methylation of the HOXB13 promoter in DU145 cells. In this study, it was shown that DNMT3b (end EZH2 protein, which was reported to recruit methylase to the HOXB13 promoter), was downregulated by ATRA. This resulted in lower trimethylation in the critical H3K27 position in the HOXB13 promoter, indicating that ATRA’s action on HOXB13 is mediated through its influence on gene methylation. ATRA, stimulating the correct differentiation of prostate cells, thus reveals not only an important therapeutic but also a preventive action [88].

Thrombomodulin (TM) is a protein localized on the surface of endothelial cells and its main function is to activate protein C, therefore inhibiting the coagulation cascade. Immunostaining studies have shown a decreased level of TM in high-grade adenocarcinomas of the prostate and many other neoplasms. Low expression of TM correlated with high malignancy and a strong increase of metastatic capability. Research has revealed significantly lower levels of TM in LNCaP, PC-3 and DU145 than in normal PrEC cells. ATRA strongly increased the level of TM mRNA and protein in PrEC cells. However, its effect was weaker in LNCaP and PC-3, while DU145 cells showed no changed level of TM after treatment with ATRA. Caffeic acid phenethyl ester (CAPE), a natural inhibitor of NF-κB, in combination with ATRA induced expression of TM in PC-3, but still not in DU145 cells. The authors hypothesized that glycogen synthase kinase 3β (GSK-3β) might act as a negative regulator of TM in PC. They showed a dose-dependent upregulation of TM expression, which was caused by inhibitors of GSK-3β administered together with ATRA in PC-3 but not in DU145 cells. The analysis of the TM promoter region detected five distinct CpG islands, which are a target for many transcriptional factors. One of them is RARγ. In PrEC cells, the RARγ binding region is unmethylated, although in PC-3 and DU145 cells all CpG sites in this region are methylated. All cell lines were treated with a DNA-demethylating agent, 5-aza-2′-deoxycytidine (5-aza-dC). In DU145 and PC-3 cell lines, which include a hypermethylated promoter TM gene, levels of mRNA specific for this protein were increased. However, in PrEC and LNCaP cells, levels of TM were not increased as these cell lines exhibit no or only a low methylated TM promoter. Treatment with 5-aza-dC made DU145 prone to ATRA [89]. It was concluded that ATRA could reduce PC’s ability to metastasize.

Conjugated ATRA forms were also examined, to assess whether this improves its effects. PC-3 and LNCaP cell lines were treated with N1,N12-bis(all-trans-retinoyl)spermine (RASP), a conjugate of ATRA and spermine. Treatment with 10 μM RASP reduced the number of cells by 50% for LNCaP and by 70% for PC-3 cells. The same concentration of ATRA alone decreased the number of cancer cells by less than 10% for both cell lines. However, the higher concentrations of RASP could not be used, as it is a poorly soluble compound. The authors suggested, that ATRA induced mainly apoptosis while RASP, at high concentrations, caused necrosis. Before treatment with these compounds, cell lines expressed undetectable levels of RARγ mRNA and normal expression of RARα mRNA. However, the amount of RARβ was different in these lines. LNCaP cells expressed low levels of mRNA specific for this receptor and PC-3 cells expressed it in barely detectable amounts. After 24 h of treatment with 1 μM ATRA and 1 μM RASP, the level of RARβ significantly increased in PC-3 cells. The authors also wanted to study the role of RARα in response to these compounds, thus they treated PC-3 cells with siRNA for RARα. Downregulation of this receptor annihilated the effect of ATRA and RASP, resulting in undetectable levels of RARβ in PC-3 cells [86]. The results indicated that ATRA and RASP intensify their activity by stimulating the production of retinoic receptors.

A significant component of solid tumors is non-malignant cells, i.e., stroma. Some of these cells are tumor-associated macrophages (TAMs). TAMs are modified M2-macrophages, which produce immunosuppressive, pro-angiogenic and pro-metastatic factors and stimulate tumor growth. An interesting strategy of solid tumor treatment is the polarization of TAMs into pro-inflammatory, anti-cancer M1 macrophages. It is widely known that retinoids play an important role in the differentiation of myeloid cells, therefore the authors investigated the influence of ATRA on TAMs. Expression of several proteins characteristic for M2-polarized macrophages, including IL-10, IL-1β, indoleamine-pyrrole 2,3-dioxygenase (IDO) and VEGF was reduced after 24 h by 1 µM ATRA.

In TAMs, both the NF-κB and the Erk pathway are strongly upregulated [90]. ATRA inhibited the activity of NF-κB, but it did not influence the phosphorylation of Erk. The proliferation of TAMs was reduced by ATRA, but it did not induce macrophage death. TAMs treated with ATRA reversed their phenotype to M1 and started to kill PC-3 cells such as naive macrophages. However, treatment with ATRA did not reduce the inhibitory effect of TAMs on autologous T-lymphocytes, and it did not affect the migration of TAMs and T-lymphocytes. Thus, stimulation of the host’s immune system using ATRA may be an interesting promising therapeutic option [90], though more data are needed.

Cdks control the cell cycle and can participate in both activating and blocking it. In the following study, DU145 cells were treated with 0.1 μM and 1 μM ATRA for 24 h. ATRA at both concentrations significantly increased the amount of Cdk5 mRNA and proteins in a dose-dependent manner. Moreover, as Cdk5 induced cell differentiation, the authors suggested that this interaction is involved in the growth inhibition of DU145 cells caused by ATRA. In the next step, DU145 cells were treated with 1 μM and 1 μM ATRA with 1 μM roscovitine (a Cdk5 inhibitor), or roscovitine alone for 24 h. The level of p27 mRNA after ATRA treatment significantly increased, although after treatment with roscovitine or ATRA with roscovitine it did not change. ATRA treatment remarkably increased the amount of Cdk5 and p27 proteins in the cytosol and p27 protein in the nucleus. ATRA reduced the proliferation of DU145 cells, and this effect was abolished by roscovitine and siRNA targeted to Cdk5. An increase in the quantity of Cdk5 induced differentiation and accumulation of cells in the G1 phase of the cell cycle. The authors suggested that this was due to the increased amount of p27 in response to induced Cdk5. These results strongly suggest that ATRA induced cell cycle arrest in the G1 phase [91].

Petrie et al. showed that the concentration of ATRA at 10 nM and above caused inhibition of cell growth, while lower concentrations did not inhibit their growth. This was tested by treating ATRA at various concentrations with LNCaP, PC-3 and DU145 cells for 5 days [87]. ATRA also increases the expression of the Laxetin protein, which is produced by the luminal cells of the normal prostate but decreases in the case of cancer cells. This protein has a significant impact on the metabolism of retinoids and the interferon-dependent inflammatory response, which plays an important role in PC and significantly affects the prognosis [92].

In another study, the effect of retinol on PC-3 cells was tested. An amount of 10 µM retinol suppressed (by 79%, vs. the control) the growth of PC-3 cells, though ATRA did not reduce the growth of cancer cells. The authors suggested that retinol and ATRA inhibited cancer growth by different mechanism(s), as the opposite results were found after treating breast cancer cells with those agents, i.e., ATRA showed superior growth-inhibitory effects compared to retinol. Retinol suppressed the PC-3 adhesion by 23%, ATRA by 13%. According to this, retinol may have a stronger anticancer effect than ATRA [93].

VA is referred to a group of compounds, including not only ATRA, but also retinene, retinal and retinol. Only one study investigated the effects of VA (defined as ATRA + retinene + retinal + retinol) against PC in the last decade. In this study by Sha et al., various concentrations of VA (from 5 to 15 μM) were applied to PC-3 cells. The results showed a time and dose-dependent reduction of cellular growth. A 15% inhibition was achieved for 72 h treatments. The authors also investigated if the concentration of VA would act synergistically with 10 μM vitamin D (VD), which was indeed found after 24 h of treatment. Further investigation showed that VA and VD combined impacted cellular proliferation and were associated with significant changes in the levels of proteins controlling apoptosis (increased Bax mRNA) and cell cycle (decreased cyclin D1 mRNA). This was followed by a decrease in mitochondrial transmembrane potential, suggesting activation of apoptosis in the mitochondrial pathway [94]. This suggests that VA (active compounds, e.g., ATRA) shares some molecular action pathways with VD. The authors hypothesized that dimers of RAR and VDR are key to this interaction. This supports the theory that investigating distinct vitamins separately may possibly not give us an answer to the question of which ones are best suited for improving health or disease status. Such an isolated approach may lead to missing critically important biological mechanisms responsible for maintaining homeostasis. This is also supported by epidemiological studies. When studies adjusted for serum VD level or calcium intake, they showed more protective effects of different compounds (e.g., retinol or LC) than the studies that did not [111,112]. At present, searching for interactions should remain the main concern for basic sciences, in order to enable a more reasonable holistic approach for further studies in humans.

### 5.6. β-Carotene

Although BC is a precursor of endogenous retinoids and a well-known antioxidant, there are only a few studies that have investigated its molecular effects during the last 10 years. Linnewiel-Hermoni et al. tested the ability of BC to inhibit cell proliferation, focusing on AR signaling. For a wide range of concentrations (0–6 μM), BC was unable to inhibit LNCaP cellular proliferation. Its effects were so weak, that an IC_50_ for LNCaP and DU145 cells could not be calculated. Only for PC-3 cells, it was estimated as 13.0 ± 2.6 μM. Incubation with 6.5 μM BC decreased the activity of an AR-luciferase construct in LNCaP cells by about 40%, however, it did not influence PSA secretion. However, BC and LC together showed significantly stronger effects on LNCaP cellular proliferation than both carotenoids alone [71], with a combination index (CI) of 0.65 (the CI is calculated by adding the ratios of concentrations of agents used in combination to concentrations of agents used separately; CI < 1 indicates a synergy and the lower it is, the stronger is the effect).

The effects of BC were also investigated in PC-3 cells. It was shown that for concentrations of BC from 1 to 5 μM, the level of VEGF in PC-3 cells increased by about 60% after 6 h of incubation (compared to control cells). This was associated with a 4-fold increase in VEGF mRNA and a 3-fold increase in VEGF protein expression. For a range of 5–10 μM, the effect was weaker. However, after the following 6 h, all changes in VEGF excretion disappeared and there was no significant difference to the control. The impact on PC-3 proliferation was in line with findings from the VEGF measurement—for 12 h of incubation with 1–5 μM BC the proliferation was slightly increased. Only when treated with 20 μM of BC, cells showed a 20% decreased proliferation [95].

The results of these two studies are similar and indicate that BC alone could rather act in a chemopromotion fashion instead of prevention of PC. It confirms results from epidemiological studies, in which the effectiveness of BC was unclear [111,113,114,115]. In addition, concentrations of BC in human serum are approximately 10-times lower compared to in vitro studies. This suggests that BC alone should not be used to prevent PC in humans.

### 5.7. Other Carotenoids

For BC, results suggesting the small potential for the prevention of PC reports on the effectiveness of AST are more inconsistent. The interference of AST with androgen signaling was investigated by Linnewiel-Hermoni et al. In brief, for 3 days of incubation with 2–5 μM AST there was a reduction in the growth of LNCaP, equal to 0–50%, showing a linear dose-dependency for these concentrations. The IC_50_ for LNCaP, DU145 and PC-3 cells were 5.5 μM, 11.0 μM and 14.0 μM, respectively. However, even concentrations equal to doubled IC_50_ values of AST (9.6 μM) did not impact the PSA excretion in LNCaP cells [71]. This suggests that AST acts through pathways that are active in LNCaP cells but not in PC-3 or DU145 cells.

Therefore, the second in vitro study on AST investigated its role in the regulation of epigenetic changes. Yang et al. hypothesized that AST acts through activating the Nrf2 pathway, which is involved in GSTP1 activation, as well as histone deacetylases (HDACs), and also DNMTs inactivation. Surprisingly, the maximum inhibitory effect on LNCaP cell proliferation after 5 days of incubation was 40%. However, it was reached for 50 μM AST, a non-physiological concentration. Treatment with lower concentrations of AST (6.25 and 12.5 μM) did not change mRNA and protein levels of Nrf2 and GSTP1. Only a slight but significant decrease in the methylated CpG ratio in the GSTP1 (but not Nrf2) promoter was found. High concentrations of AST (12.5 and 25 μM) significantly decreased DNMT and HDAC activity, but low concentrations (6.25 μM) increased HDAC activity instead [98]. Given a similar behavior in vivo, this suggests that we can possibly exclude the Nrf-2 and GSTP1 pathway as molecular targets of AST. This study varied strongly regarding the impact of AST on LNCaP cellular viability from the previous one. However, in the study by Linnewiel-Hermoni et al. [71], cells were additionally stimulated by DHT and no DHT-negative control was made there. In human studies, even after a 3-week supplementation of 20 mg of AST per day, serum concentrations did not exceed 1 μM [116]. The same concern applies to the study by Sun et al. [117], where AST was used at an even higher concentration (50 µM was the smallest used). Despite that such a concentration cannot be achieved through dietary intervention alone, AST injection into mice (DU145 model after 2 weeks growth, 2 × 10^7^ cells inoculated) was safe and effective (~90% tumor volume reduction) against PC in this experiment, when 200 mg/kg was administered.

To investigate how AST may act in living organisms, an in vivo study on mice xenografted with PC-3 cells was conducted. Ni et al. supplemented mice with 100 mg/kg (HA group) or with 25 mg/kg (LA group) of AST. For the HA group, a very strong inhibition of tumor growth was measured 31 days after PC-3 cell injection. The authors decided to check the expression of miRNA in tumor tissue of treated and untreated mice. Among 84 different miRNAs, two showed more than a 1.5-fold increase in the HA group. These were miR-375 (1.9-fold increase) and miR-487b (2.1-fold increase) [118]. As the miR-487b was shown to be a potent inhibitor of PC-3 cells (causing cell cycle arrest and increased apoptosis), it is possible that AST may act mainly through a miR-dependent pathway in PC [119]. Thus, while some anti-cancer activities of AST appear present, both in vitro and in vivo studies implemented massive doses of AST to obtain such results. Still, these doses were not reported to be toxic or harmful for the animals [120]. When administering such a dose, AST would mainly target miR-375 and miR-487b but not DNMTs or HDACs.

Additional investigations with other carotenoids were carried out (Table 5). These included fucoxanthin, phytoene/phytofluene, lutein, torulene, torularhodin and neurosporene and violaxanthin. Torulene and torularhodin induced similar changes as crocin in pro- and anti-proliferative proteins, being effective in reducing the growth of PC-3 xenografts in nude mice. Torularhodin applied at a dose of 18 mg/kg daily for 2 weeks caused a 76% tumor mass reduction. It was followed by a significant increase in Bax and CASP 3, 8, 9 expression, as well as decreased Bad [79]. In another study, phytoene/phytofluene, colorless carotenoids present in tomatoes, showed a mild antiproliferative activity by themselves, however synergizing strongly with LC, reaching a CI equal to 0.13 against LNCaP cells (while BC combined with LC resulted in a CI of 0.65) [71]. Administered together with the conventional chemotherapeutics doxorubicin and temozolomide, lutein exerted some synergistic effect. Yet, it did not change the effectiveness of paclitaxel, 15dPGJ_2_, pioglitazone and ciglitazone. An amount of 10 μM of lutein induced changes in the level of numerous proteins, causing a more than 2-fold increase in KLK8, IGF-2, TGF-β3, as well as NR5A2 and simultaneously a 3-fold decrease in KLK14, KLK15, fibroblast growth factor 7 (FGF7), MAPK15, NR0B2 and PTEN [96]. Both proliferation stimulating and proliferation inhibiting proteins changed their levels. Apart from the fact that relatively high concentrations of this agent decreased cellular viability by only 15%, it seems that changes in the protein profiles counterbalanced one another.

## 6. Carotenoids and Prostatic Physiology and Pathology Other Than PC

### 6.1. Lycopene

#### 6.1.1. Prostatic Hyperplasia (PH)/Benign Prostatic Hyperplasia

LC is present mainly in the all-trans form, but interestingly, there is a prevalence of its cis isomers in either benign or malignant prostate tissues. Whether this cis-lycopene is the more biologically active form is not known. A recent investigation was conducted to explore the inhibiting effects of cis/trans isomers of LC on the development of PH in mice. In total, 90 mice were randomly divided into nine groups (10 mice/group). The animals received different daily doses of both LC isomers as an emulsion administered by gastric gavage in soybean oil and subcutaneous injections of testosterone propionate used to induce BPH. Three groups of animals, used as a control, received either saline, finasteride and pure emulsion with soybean oil as vehicle control. After 30 days, blood samples were taken, the mice were sacrificed, and prostates were dissected for histopathologic examination. This revealed that both oral administration of all-trans and cis-isomers attenuated testosterone-induced PH. Cis-lycopene markedly reduced the levels of serum testosterone, DHT and prostate acid phosphatase (PAP). The decrease observed in the all-trans lycopene groups as compared to the cis-isomer group was smaller, but still significant [123].

LC may be combined with *Serenoa repens* (SeR) and selenium (Se). SeR extract consists of substances with antiandrogenic action, an anti-inflammatory effect and an antiproliferative proapoptotic effect, mediated through the inhibition of growth factors [124], whereas Se is an essential micronutrient present in certain antioxidant enzymes such as SOD. Treatment with the LC-Se-SeR combination was more efficient than applying only SeR in preventing BPH, and it inhibited rat prostate growth by 83%, suggesting that Se and LC at pharmacological doses potentiate SeR proapoptotic efficacy for BPH. The molecular effects of an LC-Se-SeR combination included downregulation of Bcl-2, upregulation of Bax and induction of CASP9 [123].

Another study focused on levels of inhibitor of apoptosis proteins (IAPs)—direct inhibitors of CASPs—after combined therapy with LC-Se-SeR and each compound alone. The levels of proteins, i.e., a cellular inhibitor of apoptosis protein 1 (cIAP-1), cIAP-2, nuclear inhibitor of apoptosis protein (NIAP) and BIRC5 (survivin) in rats with experimental testosterone-dependent BPH were measured. SeR, Se and LC, either alone or in combination, did not modify cIAP-1 and cIAP-2 expression, but significantly reduced NIAP and BIRC5 expression. The decrease of NIAP and BIRC5 was pronounced after LC treatment, but the most profound after application of the LC-Se-SeR combination [125]. Interestingly, NIAP is present only in either benign or malicious overgrowth of the prostate and not in normal prostate cells, suggesting that IAP might play a role in these conditions [126].

#### 6.1.2. Lycopene Metabolism, Impact on Prostate Physiology and Relation with Serum Testosterone

LC may exert multiple molecular effects. To a various extent, it may prevent oxidative DNA damage, induce phase II enzymes, decrease levels of proinflammatory cytokines, inhibit androgen activation and signaling, inhibit IGF-I signal transduction, inhibit Wingless-related integration site (Wnt)/β-catenin signaling, increase gap-junctional communication and interfere with growth factor signaling pathways, leading to cell cycle arrest and apoptosis induction [127]. Changes in testosterone levels exert an influence on LC metabolism. Castration increases hepatic LC in rats, whereas higher testosterone leads to reduced LC accumulation [128]. Further literature data suggest that a high intake of tomato phytochemicals and higher serum LC may reduce serum testosterone [129].

In a recent study, BCO1 knockout or WT mice received either a 10% tomato powder, a lycopene-containing (248 nmol/g) diet, or a control diet for 4 days, after which serum and testicle testosterone were measured. BCO1-/- mice fed with tomato powder, as well as those fed with LC, had decreased levels of both serum and testicular testosterone. The testosterone levels in WT mice did not change. The mechanism by which BCO1 knockout affects LC concentration and reduces testosterone levels remains unclear. A probable explanation emerges from the fact that expression of BCO2 is higher in BCO1-/- mice. BCO2, known to be responsible for eccentric cleavage of acyclic carotenoids (including LC) to form apo-carotenals, may metabolize LC to products, which exert their therapeutic effect by decreasing testosterone levels [128]. The key role of BCO2 in the metabolism of acyclic non-provitamin A carotenoids, such as LC, should be taken into consideration, as tomato-fed mice with BCO2 knockout had increased serum and tissue concentrations of LC [130].

#### 6.1.3. Anti-Inflammatory Properties and Signal Transduction

In an attempt to explore the impact of LC on PC or prostate hyperplasia, PrEC was treated either with LC at a physiologically relevant concentration (2.0 µM) or placebo for 48 h and then lysed and fractionated. The obtained proteins were trypsinized and derivatized [131]. The authors of this comprehensive study, adopting a multi-dimensional approach, examined various effects of LC. Exposure to LC impaired proliferation of PrEC by downregulating the Akt/mTOR pathway and by upregulating genes with growth inhibitory effects. Exposure also altered several signaling pathways, e.g., inhibiting androgen signaling, downregulating TNF-α signaling, and deactivating the MAPK pathway.

#### 6.1.4. Cytoprotection, Redox Homeostasis, Apoptosis

LC’s impact on proteins associated with apoptosis is shown in Table 6. GSTs are a family of enzymes that play an important role in detoxification by catalyzing the conjugation of many hydrophobic and electrophilic compounds with reduced glutathione [132]. Some findings suggest that LC can elevate levels of phase II enzymes that can prevent cytotoxicity due to xenobiotic electrophiles and carcinogens. In this study [131], both glutathione-S-transferase omega 1 (GSTO1) and GSTP1 were upregulated by 11% and 17%, respectively, in PrECs treated with LC. Surprisingly, contrary to the aforementioned results, treatment of PrEC cultures with LC for 48 h did not evoke any observable apoptosis.

Hydrophobic carotenoids such as LC do not possess any electrophilic group and are unlikely to directly activate the Nrf2 and the EpRE/AnRE system. Therefore, it is rather the carotenoid oxidation products, such as their BCO1/2 cleavage products and further metabolites, that are the active mediators of the EpRE/AnRE system [133]. Oxidized derivatives of carotenoids can be found both in tomatoes and in human serum and tissues. They can be formed either by spontaneous oxidation, or as a result of chemical or enzymatic catalyzed oxidation.

### 6.2. Other Carotenoids

It was established earlier that BCO1 disruption impacts diverse physiological endpoints independent of dietary carotenoid intake, including the expression of genes controlling androgen metabolism. Mice lacking BCO1 exhibited reduced serum testosterone, prostatic AR signaling, and prostatic cellular proliferation. Analysis of prostatic morphology revealed decreases in gland weight and tissue testosterone concentration. Expression of the Ki-67 proliferation marker in BCO1-/- prostate tissue was distinctly reduced, corresponding to the aforementioned morphological changes. Expression analysis of 200 PC and androgen-related genes suggested that BCO1 loss significantly disrupted prostatic AR signaling, cell cycle progression, and proliferation [22].

Some authors decided to study other carotenoids. For instance, Chao Du et al. focused on the antioxidant effects of torulene and torularhodin. According to their findings, these compounds protect human prostate stromal cells from H_2_O_2_-induced oxidative stress damage via regulating Bcl-2/Bax mediated apoptosis. Moreover, pretreatment with torulene and torularhodin distinctly impaired H_2_O_2_-induced apoptosis in human prostate stromal cells (WPMY-1) through the scavenging of intracellular ROS and inhibition of malondialdehyde overproduction, as well as the activation of catalase (CAT), SOD and glutathione peroxidase (GPx) [134].

AST is another compound that was employed to reduce oxidative stress. In a study involving prostate epithelial cells (RWPE-1) and PC cells (PC-3), Chinese researchers tried to determine AST’s effects on oxidative stress induced by Cu^2+^ ions [135]. Cu^2+^ triggered apoptosis and accumulation of intracellular ROS and malondialdehyde in both cell lines. The addition of AST solutions could decrease MDA levels, increased mitochondrial membrane potential, and kept ROS stable in RWPE-1 cells. AST decreased SOD, Gpx and CAT activity in a PC-3 cell line treated with Cu^2+^. Interestingly, an opposite effect was observed in RWPE-1 cells, suggesting that AST’s protective properties in prostate epithelial cells go hand in hand with its disturbance of the antioxidant enzyme system in PC cells.

Retinoid derivatives of provitamin A carotenoids exert multiple molecular effects and play an important, but a somewhat vague role in the development and the physiology of the prostate gland. It was shown that androgens determine the development of urogenital sinus (UGS) into the prostate and bulbourethral gland in male mammals [136]. However, other molecular pathways involved in the initiation of prostate development are still poorly understood. According to a recent study, sex-specific ATRA signaling is required for the initiation of UGS bud development in mice. ALDH catalyzes the final step in ATRA synthesis. Enzymes from this group have restricted areas of expression in the urogenital mesenchyme (UGM), which surrounds the epithelium within the UGS of male embryos at the early stages of prostate development. As confirmed by reverse transcription-polymerase chain reaction (RT-PCR), Aldh1a1 and Aldh1a3 expression were sex-specific. They were undetected in the female UGS, while Aldh1a2 was present in both males and females. Moreover, Aldh1a1 and Aldh1a3 showed a rather peri-urethral expression pattern at the epithelial–mesenchymal boundary within the male UGM. Such a correlation suggested that ATRA might play a role in prostate development initiation. In an ex vivo organ culture assay with UGS from female mice embryos, the addition of DHT proved a prerequisite to induce prostate bud formation and expression of Nkx3.1 and Sox9, early markers of prostate development. However, female UGS cultured with DHT and DEAB (4-diethylamino-benzaldehyde), an inhibitor of ALDH enzymes, had a distinctly reduced number of buds along with a severe decrease in prostate development marker expression. The addition of ATRA to UGS cultures with DHT and DEAB reversed the aforementioned effect and reactivated the development of buds. The role of ATRA receptors was challenged using a pan RAR inverse agonist, BMS493. As expected, this impaired the formation of prostate buds [137].

Another example of androgen and ATRA cooperation emerged from a study focusing on human prostatic transglutaminase (hTGP) prostate-restricted gene regulation. TGP in rodents is related to fertilization and reduction of sperm antigenicity. In humans, hTGP expression corresponds to the invasive potential of PC cells. To investigate the impact of ATRA, the prostate cell lines LNCaP, PC346C, PNT1A and PNT2C2 were treated with 500 nM ATRA. LNCaP and PC346C cancer cells treated with ATRA showed a marked increase in hTGP expression, whereas the non-tumorigenic prostate cell lines PNT1A and PNT2C2 showed a small decrease. RARγ knockdown with siRNA targeting specifically RARγ m-RNA had a significant negative effect on basal hTGP mRNA expression and its levels in ATRA-treated cells. Consequently, it is probably RARγ that plays the major role in ATRA-dependent hTGP expression. The presence of AR, but not its activity, facilitated hTGP expression. Knockout of AR in LNCaP cells, both in untreated conditions and 24 h after ATRA treatment (500 nM), decreased hTGP expression. However, inhibition of ARs’ activation by bicalutamide had no effect on hTGP levels in LNCaP cells [138].

Lecithin: retinol acyltransferase (LRAT) is the major enzyme involved in retinol esterification in most tissues. Both LRAT and RA receptor 2 (RAR2) mRNA levels were higher in normal PrEC than in the PC-3 cell line. In accordance with a hypothesis that increasing LRAT expression can potentially reduce prostate tumor progression, combination therapies that increased the expression of both RARs and GATA TFs were set up. The study revealed that the 172-bp sequence from 14 to 186 in the human LRAT promoter contained essential regulatory elements required for LRAT transcription. PrEC and PC-3 were co-transfected with RARs and GATA-4, an RA-inducible GATA TF. The pLRAT186 human LRAT promoter–reporter construct was used to determine levels of LRAT. It was found that RA receptors and GATA TFs cooperated in response to ATRA and upregulated LRAT transcription in both PrEC and PC-3 cells [139].

Ethanol alters plasma retinol concentrations proportionally to its amount consumed, but it does not change the retinol concentration in the rat prostate. However, high consumption of ethanol increased the concentration of ATRA in plasma/prostate tissue and especially induced RARβ and RARγ in the dorsal prostate lobe. Ethanol consumption and increased ATRA levels did not affect cell proliferation and apoptosis in the prostate [140]. Both synthesis and catabolism of ATRA were modulated by ethanol consumption dose-dependent. CYP26A1 and CYP26B1 are responsible for ATRA catabolism. Ethanol reduced the activity of the aforementioned CYPs and increased ATRA concentration in the prostate. It also changed the levels of ALDHA1, ALDHA2 and ALDHA3, either elevating or decreasing their concentrations in different parts of the rat prostate [141].

## 7. Conclusions

This review presents insight into the recent findings on the influence of carotenoids and retinoids on prostate physiology and pathology, with special concern given to PC and PH. To find a link between the results in observational studies and the basic biology of PC, we reviewed many laboratory studies, including cell-culture and animal models. Many promising molecular targets for carotenoids were revealed, e.g., the IGF pathway and BCO polymorphisms for LC or HOXB13 for ATRA, indicating that the assessment of variants of genes coding for those proteins might be crucial for an effective PC therapy with carotenoids. Simultaneously, a small efficacy of BC was shown, supporting as well as explaining epidemiological findings.

The profound knowledge of the metabolism of various carotenoids and their derivatives would be associated with a deeper understanding of their effects on cellular receptors and signaling pathways, one of the keys to the development of a cutting-edge approach to the prophylaxis and treatment of prostate diseases, first and foremost PC—a severe threat to the health and life of millions of men in the world, which still poses a therapeutic challenge. The diversity of carotenoids and their influence on the human organism and prostate in particular still remains a source of fascinating, surprising findings. Undoubtedly, numerous discoveries in this field are awaiting us in the following years.

## Figures and Tables

**Table 1:** Overview of carotenoids found in the diet.

Carotenes	Xanthophylls
Carotene ^1^	Lutein
	Zeaxanthin
Lycopene	Neoxanthin
	Violaxanthin
Phytofluene	Flavaxanthin
	α-Cryptoxanthin
Torulene	β-Cryptoxanthin

^1^ Forms including α, β, γ, δ, ε and ζ.

**Table 2:** Nuclear receptors involved in the metabolism of carotenoid metabolites [44].

Type of Action	Binding Partners	Mechanism
permissive	FXR, LXR, PPAR	Ligand binding to each partner facilitates nuclear co-activator (NCoA) recruitment to promote gene expression. Binding of the second NR ligand would enhance this effect.
non-permissive	TR, VDR	Binding of ligand to RXR-dimerizing partner determines its ability to recruit NCoA to facilitate gene expression. Binding of the RXR ligand would not enhance this effect.
conditionally permissive	RAR	RAR ligand binding is a necessary condition for facilitating gene expression, but it also permits the binding of RXR agonists. RXR ligand binding would enhance transcriptional response.

**Table 4:** The changes in levels of given proteins or mRNA induced by the administration of distinct carotenoids, based on cellular or animal trials. Interactions between listed factors are shown in Figure 2.

Carotenoid or Metabolite	Increased	Decreased	References
Lycopene	CASP9, Fas, HIF1α, NF-κB subunit 2, SOCS2, SKI, STAT3, STAT6	ECE1, ICAM1, IL-18, MMP9, NF-κB, TGF-β1	[74]
	ABCA1, LXRα, PPARγ	HMG-CoAR	[64]
	ABCA1, ApoAI, LXRα, PPARγ	-	[65]
	ABCA1, LXRα, p21, p27, p53, PPARγ, Bax	Akt2, Bcl-2, Bcl-XD, cyclin D1, PI3K, p-Akt, p-Erk1/2, p-JNK, p-p38, NF-κB, Ras	[77]
	Bax, CK18	Akt2	[68]
	-	Bcl-2	[78]
	Bax	-	[79]
	IGF-1	-	[80]
	p-IκB	TNF-α	[72]
	BCO2	-	[62]
	PF-4, CD41	-	[83]
	-	Aldh1a1, Ngfr, paxillin, Srd5a1, Srd5a2, Srebf1	[18]
	CASP3	-	[73]
	-	Cdk7, EGFR, PSA TGF-β2	[69]
	IGF-BP3	IGF-1R	[67]
β-Carotene	VEGF	-	[95]
All-trans-retinoic acid	HOXB13	-	[88]
	thrombomodulin	-	[89]
	RARβ	-	[86]
	FasL, MHC I, NF-κB	IDO, IL-1β, IL-10, VEGF	[90]
	Cdk5, p27	-	[91]
	Laxetin	-	[92]
	Bax	cyclin D1	[94]
	Bax, CASP3, CASP7, Fas, FADD, smac/diablo	Bcl-2, BIRC2, BIRC5, cyclin D1, LTβR, MLC-1, p53	[84]
Lutein	IGF-2, KLK8, TGF-β3	FGF7, KLK14, KLK15, MAPK15, NR0B2, PTEN	[96]
Astaxanthin	DNMTs, GSTP1, HDACs, Nrf2	-	[97]
Fucoxanthin	Bax, CASP3, 8, 9, p21, p27	Bcl-2, cyclin B1, cyclin D1, cyclin E	[98]
Torulene	Bax, CASP3, 8, 9	-	[79]
Torularhodin	Bax, CASP3, 8, 9	Bcl-2	[79]

**Table 5:** Summary of results of laboratory studies investigating the impact of additional carotenoids (other than lycopene and β-carotene) on PC cell lines.

Carotenoid	Investigated Entity	Concentration or Dose	Investigated Feature	Results	Commentary	Reference
Fucoxanthin	LNCaP	4.5 μM 2.5 μM 3.8 μM	cell viability cells in G1/phase GADD45A_mRNA_ GADD45B_mRNA_ p-JNK p-p38 p-Erk1/2	↓ (−80%) ↑ (+16.2%) ↑ (3.0 x) N/C ↑ N/C ↓	IC_50_ ~ 2.5 μM (3 days of treatment)	[121]
	LNCaP DU145 PC-3	0.55 μM 0.84 μM 0.95 μM 1.00 μM	IC_50_ IC_50_ IC_50_ p21, p27, Bax, CASP3, 8, 9 cyclin B1, cyclin D1, cyclin E, Bcl-2	0.55 μM 0.84 μM 0.95 μM ↑ ↓	All data for 48 h treatment.	[98]
Phytoene or Phytofluene	LNCaP	5.2 μM 4.0 μM 0.8 μM	IC_50_ PSA synergism with LC (0.3 μM)	5.2 μM *p* > 0.05 CI = 0.13	In each experiment, cells were additionally stimulated with 1 nM DHT.	[71]
	PC-3	9.0 μM	IC_50_			
Lutein	PC-3	10.0 μM	cell’s viability synergy ^1^ synergy with doxorubicin (0–100 μM) synergy with temozolomide (0–100 μM)	↓ (−15%) N/C (+) for 10–25 μM doxorubicin (+) for 50–100 μM temozolomide	Pioglitazone and 15dPGJ_2_ combined with lutein showed mildly increased cell death.	[96]
Torulene	PC-3 mice xenografts	18 mg/kg *p.o.* for 14 days after 49 days	tumor mass Bax, CASP3, 8, 9 Bcl-2	↓ (−72%) ↑ N/C	For each protein level, measurement torularhodin was significantly more effective than torulene.	[79]
Torularhodin	PC-3 mice xenografts	18 mg/kg *p.o.* or 14 days after 49 days	tumor mass Bax, CASP3, 8, 9 Bcl-2	↓ (−76%) ↑ ↓		
Carotenoids isolated from *Kocuria* strain QWT-12	PC-3 DU145 LNCaP	0.5–0.8 mg/mL	cell’s viability	no effect	Carotenoids produced by *Kocuria* strain were neurosporene (dominating) and violaxanthin.	[122]

^1^ With paclitaxel, 15dPGJ_2_, ciglitazone and pioglitazone within concentrations from 0 to 100 μM. Arrow up (↑) means that given entity (i.e., cell growth, apoptosis, protein concentration, or gene expression) increased (if specified, by N%, or N-times (N x)), while arrow down (↓) refers to its decrease in the analogous way.

**Table 6:** The influence of lycopene on the expression of proteins involved in the process of apoptosis [131].

**Lycopene’s Effect on Proteins Associated with Apoptosis Induction**	
Tyrosyl-tRNA synthetase (TyrRS) 40S ribosomal protein S3 (RPS3) Pyruvate kinase isozyme M2 (PKM2)	↑15–20%
**Lycopene’s Effect on Antiapoptotic Proteins**	
Chloride intracellular channel protein 1 (CLIC1)	↓35%
Heat shock 70 kDa protein (HSP70) 1A/1B HSPb1 (HSP27) Rho GDP-dissociation inhibitor 1 (Rho GDI 1) Translationally controlled tumor protein (TCTP) Lactoylglutathione lyase 78 kDa glucose-regulated protein (Grp78) Protein kinase C inhibitor protein 1 (KCIP1)	↓10–15%

## Supplementary Materials

The following are available online at https://www.mdpi.com/article/10.3390/antiox10040585/s1. PAGE ADRESS, Figure S1: The flow chart summarizing the process of data extraction.

## Abbreviations

5-aza-dC—5-aza-2′-deoxycytidine, 15dPGJ_2_—15-deoxy-Δ12,14-prostaglandin J2, ABCA1—adenosine triphosphate-binding cassette transporter subfamily A member 1, ADH—alcohol dehydrogenase, AL—algal lycopene, ALDH—aldehyde dehydrogenase, AR—androgen receptor, ARE—androgen responsive element, ASAP—atypical small acinar proliferation, AST—astaxanthin, ATBC—Alpha-Tocopherol, Beta-Carotene Cancer Prevention (study), ATRA—all-trans-retinoic acid, AnRE—antioxidant responsive element, ApoAI—apolipoprotein AI, BC—β-carotene, BCO1 or 2—β-carotene 15, 15′-oxygenase 1 or 2, BIRC2—Baculoviral IAP repeat-containing protein 2, BPH—benign prostatic hyperplasia, Bax—Bcl-2-associated X protein, Bcl-2—B-cell lymphoma 2, CAPE—caffeic acid phenethyl ester, CAR—constitutive androgen receptor, CASP—caspase, CAT—catalase, CCP—carotenoid cleavage product, CD—cluster of differentiation, CI—combination index, CK18—cytokeratin 18, CMV—cytomegalovirus, COX-2—cyclooxygenase 2, CRABP-II—cellular retinoic acid binding protein-II, CRPC—castration-resistant prostate cancer, CYPB1—cytochrome B1, Cdk—cyclin-dependent kinase, DBD—deoxyribonucleic acid binding domain, DEAB—4-diethylamino-benzaldehyde, DHT—dihydrotestosterone, DNA—deoxyribonucleic acid, DNMT3b—DNA methyltransferase 3b, DRE—digital rectal examination, ECE1—endothelin converting enzyme 1, EGFR—epidermal growth factor receptor, ELOVL2—elongation of very long chain fatty acids protein 2, ER—estrogen receptor, EpRE—electrophile-response element, FABP5—fatty acid binding protein-5, FADD—Fas-associated protein with death domain, FGF—fibroblast growth factor, FXR—farnesoid X receptor, FasL—Fas ligand, GS—Gleason score, GSK-3β—glycogen synthase kinase 3β, GSTO1—glutathione-S-transferase omega 1, GSTP1—glutathione S-transferase P1, Gpx—glutathione peroxidase, HDAC—histone deacetylase, HDL—high-density lipoprotein, HGPIN—high grade prostatic intraepithelial neoplasia, HIF1α—hypoxia-inducible factor 1-α, HMG-CoAR—hydroxymethylglutaryl-CoA reductase, HMOX-1—heme oxygenase 1, HNF4—Hepatocyte nuclear factor 4, IC_50_—half-maximal inhibitory concentration, ICAM1—intercellular adherence molecule 1, IDO—Indoleamine-pyrrole 2,3-dioxygenase, IGF-1—insulin-like growth factor 1, IGF-BP3—insulin-like growth factor binding protein 3, IGF-IR—insulin-like growth factor-I receptor, IL—interleukin, IκB—inhibitor of kappa B, JNK—Jun N-terminal kinase, KLK—kallikrein, Keap1—kelch-like ECH-associated protein 1, LB—lycopene beadlets, LBD—ligand binding domain, LC—lycopene, LDL—low-density lipoprotein, LRAT—lecithin:retinol acyltransferase, LTβR—lymphotoxin beta receptor, LXR—liver X receptor, MCL-1—myeloid cell leukemia 1, MENS—The Molecular Effects of Nutritional Supplement, MHC—major histocompatibility complex, MMP9—matrix metalloprotease 9, MOOSE—Meta-analysis of Observational Studies in Epidemiology, MSK1—mitogen- and stress-activated protein kinase 1, MTTP—microsomal triglyceride transfer protein, NCoA—nuclear co-activator, NCoR—nuclear co-repressor, NF-κB—nuclear factor kappa-light-chain-enhancer of activated B cells, NFE2L2—Nuclear Factor Erythroid 2 Like 2, NIAP—nuclear inhibitor of apoptosis protein, NICE—National Institute for Health and Clinical Excellence, NLS—nuclear localization signal, NOR—nuclear orphan receptor, NOS—Newcastle-Ottawa Scale, NR—nuclear receptor, Nrf2—nuclear factor E2-related factor 2, Nurr1—Nuclear receptor related 1 protein, PAP—prostatic acid phosphatase, PC—prostate cancer, PF-4—platelet factor-4, PH—prostatic hyperplasia, PI3K—phosphatidylinositol 3-kinase, PPAR—peroxisome proliferator-activated receptor, PSA—prostate specific antigen, PTEN—phosphatase and tensin homolog, PTM—post-translational modification, PXR—pregnane X receptor, PoS—placebo sera, RAE—retinol activity equivalent, RARE—Retinoic Acid Receptor Responsive Element, RARα—retinoic acid receptor α, RASP—N1,N12-bis(all-trans-retinoyl)spermine, RBP—retinol binding protein, RCT—randomized controlled trial, ROR (or NR1F)—retinoid orphan receptor, ROS—reactive oxygen species, RT—red tomato paste, RT-PCR—reverse transcription polymerase chain reaction, RTS—red tomato sera, RXR—retinoid X receptor, RXRE—Retinoid X Receptor Responsive Element, Rho-GTP-ase—Ras homolog family member guanosine triphosphate hydrolase, SCARB1—scavenger receptor class B type 1, SCNC—small cell neuroendocrine carcinoma, SDR—short-chain dehydrogenase, SKI—TGF-β signaling repressor, SNP—single nucleotide polymorphism, SOCS2—suppressor of cytokine signaling 2, SOD—superoxide dismutase, STAT—signal transducer and activator of transcription, SeR—*Serenoa repens*, TAM—tumor-associated macrophage, TC—tomato LC, TF—transcription factor, TGF-β—transforming growth factor beta, TM—thrombomodulin, TNFRSF—tumor necrosis factor receptor superfamily, TNF-α—tumor necrosis factor α, TP—tomato powder, TP53—tumor protein 53, TR3—triiodothyronine receptor, TRAMP—Transgenic Adenocarcinoma Mouse Prostate, UGM—urogenital mesenchyme, UGS—urogenital sinus, VA—vitamin A, VCAM—vascular cell adhesion molecule, VD—vitamin D, VDR—vitamin D receptor, VEGF—vascular and epithelial growth factor, WT—wild-type, Wnt—Wingless-related integration site, YT—yellow tomato paste, YTS—yellow tomato sera, bZIP—basic leucine zipper, cDNA—complementary DNA, cIAP-1—cellular inhibitor of apoptosis protein 1, hTGP—human prostatic transglutaminase, mRNA—messenger ribonucleic acid, mTOR—mechanistic target of rapamycin, miRNA—micro RNA, p38-MAPK—p38-mitogen-activated protein kinase, sMaf—small musculoaponeurotic fibrosarcoma, siRNA—small inhibitory RNA, tRXR—truncated RXR, ω3-PUFA—ω3-polyunsaturated fatty acid.
