# Supplementary material for: Recent Progress in Discovering the Role of Carotenoids and Their Metabolites in Prostatic Physiology and Pathology with a Focus on Prostate Cancer—A Review—Part I: Molecular Mechanisms of Carotenoid Action
Source: Antioxidants (Basel). 2021 Apr 10;10(4):585. doi: 10.3390/antiox10040585 (PMC8069951; doi:10.3390/antiox10040585)
Supplement: Supplementary file 1 [file antioxidants-10-00585-s001.pdf]

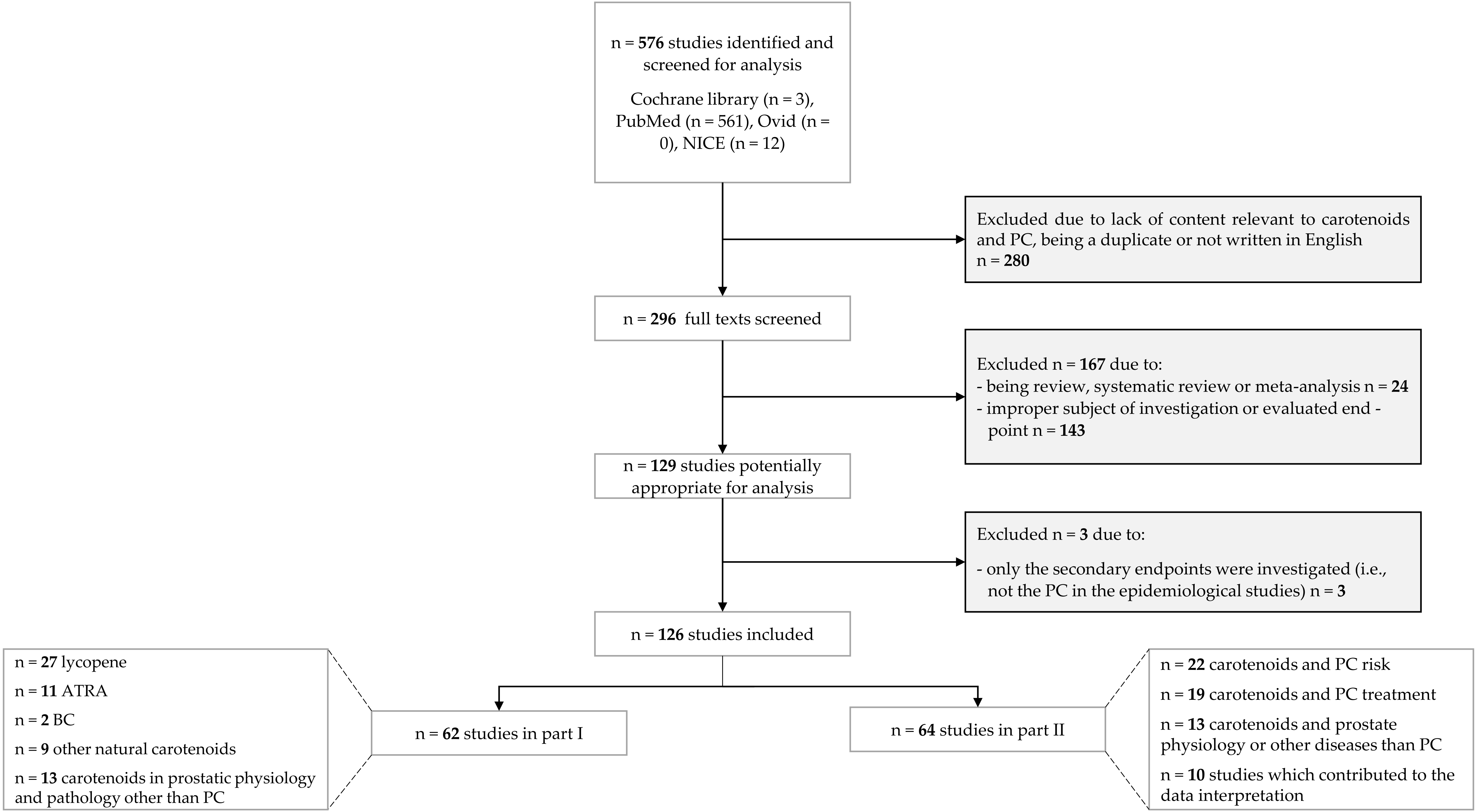
n = 576 studies identified and screened for analysis
Cochrane library (n = 3), PubMed (n = 561), Ovid (n = 0), NICE (n = 12)
Excluded due to lack of content relevant to carotenoids and PC, being a duplicate or not written in English n = 280
n = 296 full texts screened
Excluded n = 167 due to:
- being review, systematic review or meta-analysis n = 24
- improper subject of investigation or evaluated end - point n = 143
n = 129 studies potentially appropriate for analysis
Excluded n = 3 due to:
- only the secondary endpoints were investigated (i.e., not the PC in the epidemiological studies) n = 3
n = 126 studies included
n = 27 lycopene
n = 11 ATRA
n = 2 BC
n = 9 other natural carotenoids
n = 13 carotenoids in prostatic physiology and pathology other than PC
n = 62 studies in part I
n = 64 studies in part II
n = 22 carotenoids and PC risk
n = 19 carotenoids and PC treatment
n = 13 carotenoids and prostate physiology or other diseases than PC
n = 10 studies which contributed to the data interpretation
